# High-Mannose *N*-Glycans as Malignant Progression Markers in Early-Stage Colorectal Cancer

**DOI:** 10.3390/cancers14061552

**Published:** 2022-03-18

**Authors:** Fanny Boyaval, Hans Dalebout, René Van Zeijl, Wenjun Wang, Arantza Fariña-Sarasqueta, Guinevere S. M. Lageveen-Kammeijer, Jurjen J. Boonstra, Liam A. McDonnell, Manfred Wuhrer, Hans Morreau, Bram Heijs

**Affiliations:** 1 Department of Pathology, Leiden University Medical Center, Albinusdreef, 2333 ZA Leiden, The Netherlands; f.f.m.boyaval@lumc.nl; 2 Center for Proteomics & Metabolomics, Leiden University Medical Center, Albinusdreef, 2333 ZA Leiden, The Netherlands; h.dalebout@lumc.nl (H.D.); r.j.m.van_zeijl@lumc.nl (R.V.Z.); w.wang@lumc.nl (W.W.); g.s.m.kammeijer@lumc.nl (G.S.M.L.-K.); m.wuhrer@lumc.nl (M.W.); 3 Department of Pathology, Cancer Center Amsterdam, Amsterdam University Medical Centers, University of Amsterdam, Meibergdreef 9, 1105 AZ Amsterdam, The Netherlands; a.farina@amsterdamumc.nl; 4 Department of Gastroenterology and Hepatology, Leiden University Medical Centre, 2300 RC Leiden, The Netherlands; j.j.boonstra@lumc.nl; 5 Fondazione Pisana per la Scienza ONLUS, Via Ferruccio Giovannini, 56017 San Giuliano Terme, Italy; l.a.mcdonnell@outlook.com

**Keywords:** colorectal cancer, *N*-glycosylation, mass spectrometry imaging, MALDI-MSI, molecular histology, early cancer progression

## Abstract

**Simple Summary:**

The detection of colorectal cancer (CRC) at an early stage is increasing due to the implementation of screening programs. Local excision of early CRC is potentially curative, however the identification of early lesions at high risk of regional metastases remains challenging, and greatly influencing therapy decision making. Variations in sugar molecules has been associated with development and progression in various cancer types including CRC. Therefore, we examined these sugar signatures, so-called *N*-glycans, in different stages of progression of CRC starting from epithelium to pre-cancerous and cancerous tissue. We report that the sugar signatures clearly differentiate each step of CRC progression, especially between pre-cancerous and cancerous tissue. We also observed some of the glycosylation signatures of the cancerous areas to be spreading into the tumor microenvironment.

**Abstract:**

The increase incidence of early colorectal cancer (T1 CRC) last years is mainly due to the introduction of population-based screening for CRC. T1 CRC staging based on histological criteria remains challenging and there is high variability among pathologists in the scoring of these criteria. It is crucial to unravel the biology behind the progression of adenoma into T1 CRC. Glycomic studies have reported extensively on alterations of the *N*-glycomic pattern in CRC; therefore, investigating these alterations may reveal new insights into the development of T1 CRC. We used matrix-assisted laser desorption ionization (MALDI) mass spectrometry imaging (MSI) to spatially profile the *N*-glycan species in a cohort of pT1 CRC using archival formalin-fixed and paraffin-embedded (FFPE) material. To generate structural information on the observed *N*-glycans, CE-ESI-MS/MS was used in conjunction with MALDI-MSI. Relative intensities and glycosylation traits were calculated based on a panel of 58 *N*-glycans. Our analysis showed pronounced differences between normal epithelium, dysplastic, and carcinoma regions. High-mannose-type *N*-glycans were higher in the dysplastic region than in carcinoma, which correlates to increased proliferation of the cells. We observed changes in the cancer invasive front, including higher expression of α2,3-linked sialic acids which followed the glycosylation pattern of the carcinoma region.

## 1. Introduction

Population-based screening for colorectal cancer (CRC) is performed in the Netherlands since 2014 [1]. This screening program has led to a significant increase in the detection of early CRC defined as pT1 colorectal cancer (T1). CRC oncogenesis is a well described multi-step process where normal epithelium becomes malignant following several molecular events. First and through the acquisition of certain mutations, the normal epithelium develops into an adenomatous lesion (dysplasia) which remains limited to the mucosa. The linear acquisition of mutations in more genes lead eventually to the development of a malignant lesion (carcinoma), which by definition invades the submucosa and beyond and can eventually spread systemically (metastasis) [2]. According to the cancer staging system published by the American Joint Committee on Cancer, only adenoma that have invaded through the mucosa into the submucosa should be considered pT1 CRC [3]. Adenoma can easily be detected during colonoscopy and most of them have long term risk of progressing into cancer [4]. The determination of baseline oncological staging, treatment and surveillance policy remains challenging as pT1 CRC are locally excised and therefore lymph nodes are not harvested. The risk of lymph nodes metastases is determined based on histological criteria. There is high interobserver variability in the determination of these high risk features [5,6]. There is an urgent need for reliable markers in the risk stratification of these patients.

In recent years, matrix-assisted laser desorption/ionization mass spectrometry imaging (MALDI-MSI) has been shown to be able to add additional molecular dimensions to conventional histopathological techniques, and which includes *N*-glycans [7,8,9]. *N*-glycans are common post-translational protein modifications and are known to play major roles in fundamental molecular and cellular processes occurring in cancer, such as tumor cell invasion, cell motility and metastasis formation, angiogenesis, and immune modulation [10,11]. Variations in glycosylation signatures have been associated with the development and progression of various types of cancer, for example myxoid liposarcoma, breast cancer, prostate cancer and CRC [12,13,14,15]. In CRC, changes in the *N*-glycome have shown to be discriminative between normal and cancerous epithelial cell lines and potentially provide tumor stage specificity [16,17,18]. Using MALDI-MSI of the *N*-glycome in a stage II CRC patient cohort, we have previously shown that the glycomic changes are discriminative between adjacent normal epithelial areas and the carcinoma. In addition, glycomic differences in the tumor microenvironment have been observed between patients with good and poor prognosis [19].

In the current study we used MALDI-MSI to evaluate the *N*-glycomic signatures of dysplastic tissue regions and early invasive carcinoma regions to gain new insights into *N*-glycomic alterations during early pT1 CRC development and progression.

## 2. Materials and Methods

### 2.1. Chemicals and Reagents

Sodium bicarbonate, glacial acetic acid (HAc), hydrogen peroxide and ethanol (EtOH) were obtained from Merck (Darmstadt, Germany). α-cyano-4-hydroxycinnamic acid (CHCA), 2-picoline borane, dimethylsulfoxide (DMSO), 1-hydroxybenzotriazole hydrate (HOBt), 50% sodium hydroxide, ammonium acetate, and trifluoroacetic acid (TFA) were obtained from Sigma-Aldrich (St. Louis, MO, USA). HPLC SupraGradient acetonitrile (ACN) was obtained from Biosolve (Valkenswaard, The Netherlands). 1-ethyl-3-(3-dimethylaminopropyl) carbodiimide (EDC) and hydrochloride acid was obtained from Fluorochem (Hadfield, UK). Recombinant PNGase F PRIME-LY™ Glycosidase from Flavobacterium meningosepticum was purchase from N-Zyme Scientifics (Doylestown, PA, USA). Peptide calibration standard II was purchased from Bruker Daltonics (Bremen, Germany). All buffers were prepared using deionized water (mQ) generated from a Q-card 2 system (Millipore, The Netherlands). Poly-horseradish peroxidase solution (Poly-HRP) was obtained from Immunologic (Duiven, The Netherlands). 3,3’-diaminobenzidine (DAB) was obtained from DAKO (Agilent Technologies, Santa Clara, CA, USA).

### 2.2. Tissue Sample Collection

For this study, tissue from consecutive patients treated with local excision of pT1 CRC (*n* = 21) was collected. Archival FFPE material was retrieved from the Department of Pathology of Leiden University Medical Center (LUMC). Histopathological features are presented in Table 1. All samples were anonymized, according to the national ethical guidelines (“Code for Proper Secondary Use of Human Tissue”, Dutch Federation of Medical Scientific Societies).

### 2.3. Sample Preparation for N-Glycan MALDI-MSI

FFPE tissues were sectioned using a microtome (Leica Biosystems RM2245 Microtome) at 6 µm thickness. Deparaffinization, rehydration, sialic acid linkage-specific derivatization and *N*-glycan release were performed as described previously [19]. Briefly, paraffin was removed by consecutive washes in xylene and then rehydrated in EtOH baths, followed by water baths. On-tissue derivatization was performed by incubating the tissues slides in derivatization solution (250 mM EDC, 500 mM HOBt and 250 mM dimethylamine in DMSO) followed by addition of a 25% ammonia solution. On-tissue enzymatic *N*-glycan release was performed using a SunCollect sprayer (SunChrom, Friedrichsdorf, Germany) applying 10 layers of PNGase F (0.1 μg/μL in Tris buffer) at 10 μL/min. *N*-glycans were released in a humid environment at 37 °C overnight. After incubation, slides were dried in a vacuum desiccator (10 min), followed by matrix application (5 mg/mL CHCA in 50:49.9/0.1 (%; *v*/*v*/*v*) ACN:mQ:TFA) using the SunCollect sprayer (6 layers at (1) 10 μL/min, (2) 20 μL/min, (3) 30 μL/min, (4+) 40 μL/min).

### 2.4. N-Glycan MALDI-MSI and Histopathological Analysis

*N*-glycan MALDI-MSI was performed in positive ion reflectron mode on a rapifleX MALDI-TOF/TOF-MS instrument (Bruker Daltonics, Bremen, Germany). Spectra were recorded over an *m*/*z* range of 900–3300 Th using 1000 laser shots per pixel with 50 × 50 µm^2^ pixel size (laser setting “*M5 small*”).

Post-analysis, excess MALDI matrix was removed from the tissues by washing the slide with 70% EtOH (2 × 5 min), and tissues were stained using hematoxylin and eosin (H&E) following routine histopathological procedures. Stained tissues were scanned using a high-resolution digital slide scanner (IntelliSite Pathology Ultra-Fast Scanner, Philips, Eindhoven, The Netherlands), and images of the stained sections were co-registered to the MALDI-MSI data in the flexImaging software. Expert pathologists (A.F.-S.; H.M.) performed a histopathological analysis on the MALDI-MSI-analyzed tissues, and annotated specific regions of interest (ROIs), which were transferred to the flexImaging software. MALDI-MSI data, co-registered H&E images and ROIs were imported into SCiLS lab software 2016b (Version 4.01.8781, Bruker Daltonics, Bremen, Germany).

### 2.5. Data Preprocessing and Analysis

The total ion count (TIC)-normalized overall average spectrum of the full dataset was exported from SCiLS Lab and loaded in mMass [20]. The spectrum was preprocessed using the following parameters: (i) baseline subtraction: precision—15; relative offset—25, (ii) Savitsky-Golay smoothing: window width—*m*/*z* 0.05; cycles—4, (iii) peak picking: signal-to-noise (S/N) threshold—≥3, and (iv) Deisotoping: maximum charge—1+; isotope mass tolerance—*m*/*z* 0.15; isotope intensity tolerance—50%.

An average spectrum for every individual ROI was exported from SCiLS Lab, and imported in MassyTools, a data preprocessing tool for targeted high-throughput *N*-glycan MALDI-MS data extraction [21]. In MassyTools, an internal recalibration on all ROI average spectra was performed based on a list of internal calibrants. Spectra with at least four calibrants present were recalibrated and kept for further processing, spectra from ROIs that did not meet this criterium were discarded from further analysis. Targeted feature extraction from the ROI average spectra was performed based on a “*composition list*” containing all previously assigned *N*-glycan compositions. The quality of the extracted data was assessed through the following criteria: (i) ROI average spectra were included when ≥ 50% of the total intensity had a S/N ≥ 9, (ii) specific analytes were included for further analysis with a mass error below 20 ppm, a S/N ≥ 9 and an isotopic pattern quality (IPQ) score ≤ 0.5 in the majority of ROI average spectra (≥50%). ROIs coming from the same patient and with the same morphology were averaged. Relative intensities of all *N*-glycans were rescaled to a total sum of 100%. Derived traits were calculated from single the *N*-glycans using an in-house developed R-script (Supplementary Information, Table S1). Each trait is defined by considering the biosynthetic pathway and specific glycosylation features (e.g., glycan type, bisection, fucosylation, sialic acid linkages, antennarity).

Statistical analyses were performed in MATLAB (R2016a, 9.0.0.341360) and contained feature selection using a Kruskal-Wallis test (*p*-value threshold < 0.05). Selected features were tested for significance using a Wilcoxon rank sum test and corrected for multiple testing using the Benjamini-Hochberg method (Supplementary Information, Table S2).

### 2.6. Ki-67 Immunohistochemistry

For immunohistochemical staining of Ki-67, tissues were cut and mounted on Starfrost adhesive microscope glass slides, deparaffinized in xylene, followed by EtOH baths (2x) and incubated in 3% hydrogen peroxide for 20 min. The tissue sections were rehydrated prior to heat-induced epitope retrieval in a citrate bath in the microwave oven (750 W) for 10 min. An 1 h incubation with blocking solution (Tris-buffered saline supplemented with 0.05% Tween (TBST)/5% normal goat serum) was performed followed by overnight incubation with the primary Ki-67 antibody (D2H10 monoclonal antibody; 1:400; Cell Signaling, Danvers, MA, USA) at 4 °C. After rinsing, the sections were incubated with 100 µL of poly-HRP (30 min), incubated with DAB (5 min) and counterstained with hematoxylin following routine procedures. Negative controls for the staining were performed on consecutive tissue sections by omitting the primary antibody. Positive controls were performed on sections from human tonsil.

QuPath was used for scoring lesional positive cells for Ki-67 [22]. On each tissue, the same ROIs were annotated compared to the MSI data. The number of positive cells in each ROI was calculated through “cell detection” using the following parameters: detection image, optical density sum; requested pixel size—0.5 µm; background radius—8 µm; median filter radius—0 µm; sigma—1.5 µm; minimum cell area—10 µm^2^; maximum cell area—400 µm^2^; threshold—0.1; max background intensity—2.

### 2.7. N-Glycan Extraction and Identification by Tandem MS

For identification by tandem MS (MS/MS) analysis, consecutive tissue sections were cut and mounted on Starfrost adhesive microscope glass slides, deparaffinized, rehydrated followed by the same enzymatic *N*-glycan release with PNGase F described above. Released *N*-glycans were extracted by distributing 100 µL mQ across the tissue and incubated at 37 °C for 15 min. Extracts were collected using a pipette, dried in a vacuum centrifuge at 50 °C and resuspended in 5 µL pure water. Subsequently, released *N*-glycans were derivatized by ethyl esterification and amidation reaction, as described previously [23]. Briefly, 1 µL of dissolved sample was added to 20 µL of ethyl esterification reagent (250 mM EDC / 250 mM HOBt in EtOH) in a 96-well PCR plate and incubated for 30 min at 37 °C. To stabilize derivatives, 4 µL of 28% ammonia in water was added to the reaction, followed by a 30 min incubation at 37 °C. The addition was stopped through the addition of 24 μL ACN. Subsequently, samples were purified and enriched by cotton hydrophilic interaction liquid chromatographic solid-phase extraction (HILIC-SPE) as described before [24]. Derivatized *N*-glycans were eluted in 10 µL pure water.

After sialic acid derivatization, released and purified *N*-glycans were labelled at the reducing end by Girard’s reagent P (GirP) as described previously, with minor modifications [25]. Briefly, 10 µL of the purified and derivatized *N*-glycans were dried in the Speedvac at 60 °C. After evaporation, the sample was reconstituted in 2 µL of GirP (50 mM GirP in 10% glacial HAc and 90% EtOH), followed by an incubation step of 1 h at 60 °C. Afterwards, the samples were dried in a vacuum concentrator for 10 min at 60 °C and resuspended in 10 µL pure water for further analysis by capillary electrophoresis electrospray ionization tandem mass spectrometry (CE-ESI-MS/MS).

### 2.8. CE-ESI-MS/MS Analysis

All CE-ESI-MS/MS analyses were performed on a CESI 8000 system (SCIEX, Framingham, MA), coupled to an Impact HD UHR-QqTOF-MS (Bruker Daltonics, Bremen, Germany) via an OptiMS Bruker MS adapter (SCIEX Framingham, MA, USA), which allowed optimal alignment between the capillary spray tip and the front of the nanoshield (Bruker Daltonics, Bremen, Germany). Additionally, the adapter was modified to enable the usage of a dopant enriched nitrogen gas supply [26] (nanoBooster technology from Bruker Daltonics, Bremen, Germany). CE separation was achieved using a neutral OptiMS cartridge (91 cm long, 30 µm i.d., 150 µm o.d.; SCIEX Framingham, MA, USA). Prior to each analysis the capillary was rinsed with 0.1 M hydrochloric acid (5 min) and the background electrolyte (BGE) of 20% HAc (10 min) at 100 psi pressure. The conductive line was rinsed with BGE for 3 min at 75 psi pressure. Before analysis, all samples were diluted with leading electrolyte (final concentration of 400 mM ammonium acetate, pH 3.17) and were hydrodynamically injected by applying a pressure of 10 psi for 60 s, corresponding to 14% of the total capillary volume (88 nL). After each sample injection, a BGE post plug was injected by applying a pressure of 2.5 psi for 15 s (0.6% of the capillary volume). For each analysis separation was achieved using 20 kV and applying a constant flow, of 2 psi, the capillary temperature was set at 20 °C.

All MS detection after CE separation were performed in positive ionization mode with a capillary voltage of 1200 V. Temperature and flow rate of the drying gas were set at 150 °C and 1.2 L/min, respectively. To minimize the in-source decay, the collision cell energy and the quadrupole ion energy were set at 7.0 eV, as well as the pre-pulse storage was set at 15.0 µs. Untargeted fragmentation was completed at 1.00 Hz on the three most abundant precursor ions in a range of *m*/*z* 150–2000 with a minimum intensity of 4548. Targeted fragmentation was performed using an inclusion list, precursor ions were selected with a minimum intensity of 600. The precursor ions were isolated with a width of 8–15 depending on *m*/*z* values. The collision energies were set as a linear curve in a *m*/*z* dependent manner, ranging from 20 eV at *m*/*z* 500 to 70 eV at *m*/*z* 2000 for all charge states (1–3), applying a basic stepping mode with collision energy (100–50%) each 80% and 20% of the time, respectively.

### 2.9. CE-ESI-MS/MS Data Processing

Raw CE-MS data were internally calibrated with Data Analysis 4.2 (Bruker Daltonics, Bremen, Germany) using a cluster of GirP adducts early migrating in the electropherogram with an intensity threshold of 1000 and a search window of 0.02 Da. The data was manually screened based on their exact mass, migration order as well as the assigned *N*-glycans from the MSI analysis. The potential *N*-glycan structures were based on the MS/MS identification and drawn in GlycoWorkBench (Supplementary Information, Table S3) [27].

## 3. Results

### 3.1. The N-Glycome of Early-Stage CRC; from Normal Epithelium to Adenoma and Carcinoma

**Figure 1:**
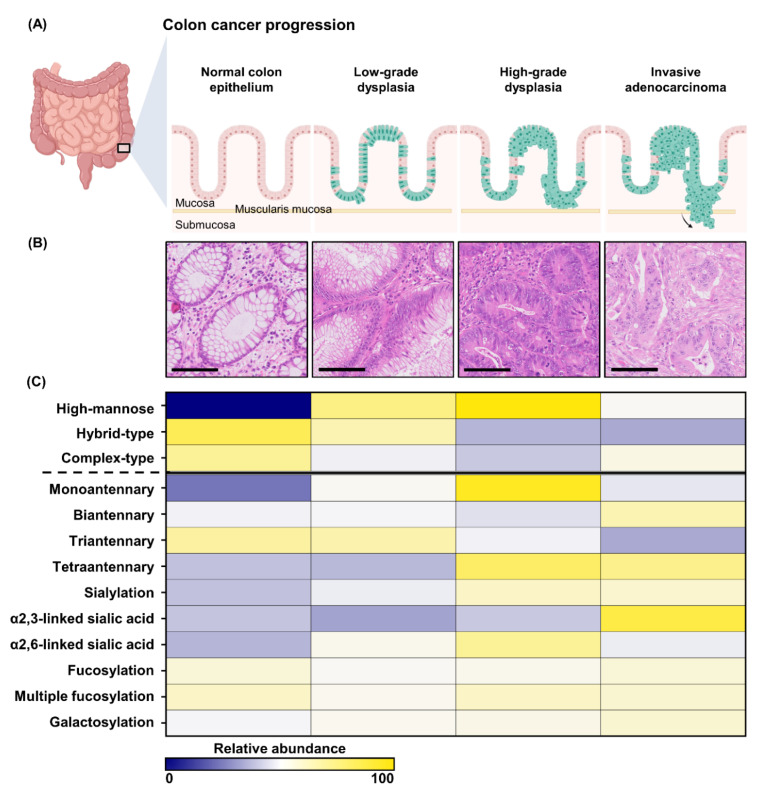
Morphological and molecular progression of CRC. (**A**) A schematic representation of the morphological progression from normal colon epithelium to dysplasia and ultimately invasive carcinoma. (**B**) Examples of the different morphological stages of CRC development (40× magnification) using H&E stained tissues. The scale bars represent 100 µm. (**C**) The molecular progression of normal colon epithelium to CRC on the glycomic level. The glycome, shown here as different *N*-glycosylation traits, change throughout the progression to CRC.

The first aim in this study was to find *N*-glycosylation patterns describing the morphological progression from normal colon epithelium to carcinoma (Figure 1A,B). *N*-glycan MALDI-MSI and a histopathological examination was performed on tissues from 21 pT1 CRC patients. In the histopathological examination specific histological ROIs were annotated: (i) adjacent normal colon epithelium (NE), (ii) low-grade dysplasia (LGD), (iii) high-grade dysplasia (HGD), and (iv) carcinoma (CA). Examples of the annotated regions can be found in Supplementary Information, Figure S1 and Supplementary Document File S1. A total of 58 *N*-glycans were detected consistently in all tissue types following the curation of the spectra (See Supplementary Information, Figure S2 and Table S3).

**Figure 2:**
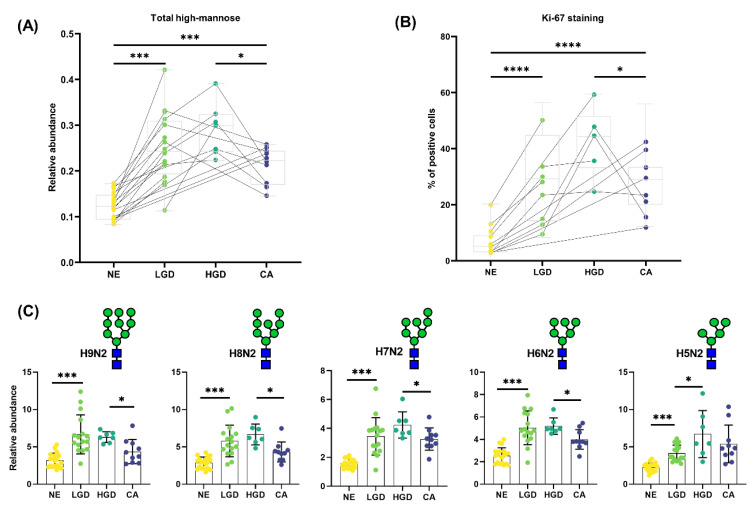
High-mannose *N*-glycans are abundant in proliferative cells. (**A**) The abundance of high-mannose-type *N*-glycans changes with progression through the morphological spectrum of T1 CRC development. (**B**) The changes in number of proliferative cells (Ki-67^+^) cells shows a similar pattern to the changes in high-mannose *N*-glycans. (**C**) All individual high-mannose-type *N*-glycans contribute and change in a similar fashion to the high-mannose trait depicted in (**A**). * = *p*-value ≤ 0.05, *** = *p*-value ≤ 0.001, **** = *p*-value ≤ 0.0001.

High-mannose-type (HM) *N*-glycans showed a very distinct pattern throughout CRC development (Figure 1C, Supplementary Figure S2) when the histology-specific analysis was explored with the derived traits. The abundance of HM-type *N*-glycans more than two-fold higher in HGD compared to NE (FC_HGD/NE_ = 2.34, *p*-value = 0.0001) and exhibited a trend towards an increase from LGD to HGD (FC_HGD/LGD_ = 1.16, *p*-value = 0.229). HM abundance was lower in CA (FC_CA/HGD_ = 0.73, *p*-value = 0.014). These results were observed in the averages of the morphological groups, in all dataset from the 21 individual patients (Figure 2A) and persisted for all individually detected HM species (Figure 2C). To assess whether the observed changes in HM-type *N*-glycans were related to the proliferation of (malignant) epithelial cells, a Ki-67 staining was performed on adjacent tissue sections from the same FFPE blocks (Figure 2B and Supplementary Information, Figure S3). The percentage of proliferating cells (Ki-67^+^) was found to be higher in HGD compared to NE (FC_HGD/NE_ = 6.10, *p*-value = 0.0003), and lower in CA compared to HGD (FC_CA/HGD_ = 0.75, *p*-value = 0.060).

In addition to changes in HM-type *N*-glycans, we observed concomitant changes in the relative abundances of complex-type *N*-glycans (Figure 1, Supplementary Figures S2 and S4). Within the large group of complex-type *N*-glycans variations were observed of specific traits with disease progression. For example, in the transition from NE to HGD a change in the distribution of complex-type antennarity was detected; while the abundance of complex diantennary *N*-glycans (CA2) remained unchanged, it appeared that the abundances of triantennary *N*-glycans were lower in HGD (CA3, FC_HGD/NE_ = 0.87, *p*-value = 0.030), and tetraantennary *N*-glycans were higher in HGD (CA4), FC_HGD/NE_ = 1.36, *p*-value = 0.022).

Additionally, the level of galactosylation in complex-type *N*-glycans (CG) was slightly higher in CA compared to NE (FC_CA/NE_ = 1.06, *p*-value= 0.001).

Sialylation (CS) appeared higher in HGD compared to NE (FC_HGD/NE_ = 1.18, *p*-value = 0.014), and remained unchanged in the progression from HGD to CA. Differences in the relative abundances of sialic acid linkage isomers were observed between the various stages of disease progression. Complex-type *N*-glycans with α2,6-linked sialic acids (CD) followed a similar pattern to the HM-type *N*-glycans, with a higher abundance in HGD compared to NE (FC_HGD/NE_ = 1.30, *p*-value = 0.006), and a lower abundance in CA (FC_CA/HGD_ = 1.39, *p*-value = 0.047). In contrast, complex-type *N*-glycans with α2,3-linked sialic acids (CAm) remained unchanged in the progression from NE to HGD and were higher in CA compared to HGD (FC_CA/HGD_ = 1.39, *p*-value = 0.047). Similar patterns were also observed when the specific sialic linkage complex-type *N*-glycans were combined with fucosylated species (CF + D and CF + Am), although no significant changes in only fucosylation were observed.

### 3.2. Glycomic Traits Visualization

**Figure 3:**
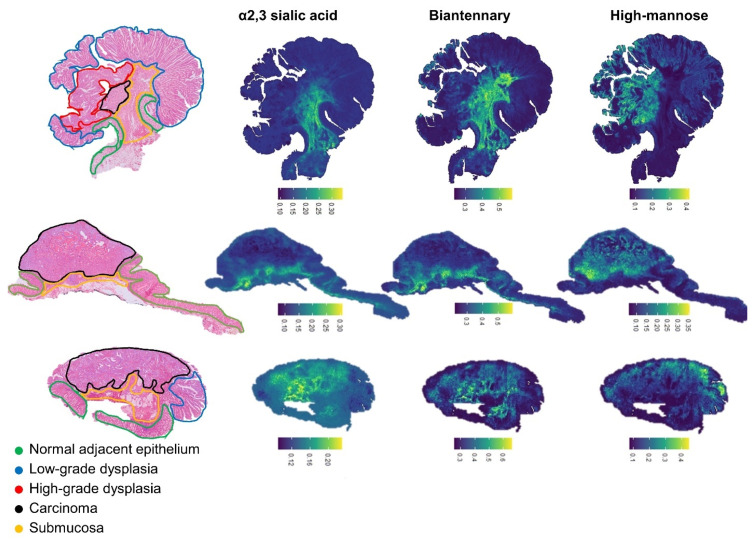
Glycosylation trait image. H&E staining of three annotated slides are depicted at the left side and the remaining images are three reconstructed derived traits in the example tissues. The scale represents the relative expression of the traits.

To assess if the change in glycomic signatures observed in the carcinoma areas were directly linked to the cellular changes due to cancer progression or an effect of the invasion of the cancer cells in the submucosa (the direct microenvironment of the cancer cells), we reconstructed glycosylation trait images to visualize their distributions in the tissue sections (Figure 3). We focused on the traits showing significant increase in CA compared to other morphologies. This led to the observation that both CA2 and CAm complex-type *N*-glycans were increased in the submucosa and in the carcinoma areas mainly close to the invasion site. On the contrary, the relative intensity of HM confirmed the pattern previously observed, namely HM exhibited higher intensities in HGD areas compared to CA when both morphologies were present. Additionally, when dysplastic regions were absent, the HM-type *N*-glycans were clearly restricted to the CA region.

### 3.3. Cancer Glycosylation Signatures of Patients with Lymph Node Metastasis

We applied a similar approach as above to compare the patient with or without lymph node metastasis (LNM) as predicting the risk of developing LNM is a major challenge and can impact treatment option. We compared the relative abundances of *N*-glycans and glycan traits between the CA areas of patient with LNM (LNM+; *n* = 5) and patient without LNM (LNM0; *n* = 4). A single *N*-glycan was differentiating the groups: the fully sialylated triantennary complex-type glycan (H6N5Am1D2, FC_LNM+/LNM-_ = 1.05, *p*-value = 0.032) was detected at higher levels in LNM+ patients (Supplementary Information, Figure S5).

## 4. Discussion

In this study, we report a comprehensive characterization of *N*-glycosylation in early CRC tissues, revealing distinct *N*-glycosylation signatures of cancer progression with potential applications in diagnostic pathology. Previous reports predominantly focused on either advanced CRC stages or on plasma samples instead of tissue samples [28,29]. The use of plasma in the latter example meant it was not possible to directly correlate any changes in *N*-glycosylation with specific histopathological information. Here we reported the identification, using a MALDI-MSI-based approach, of the *N*-glycosylation signatures of adenoma and early carcinoma of CRC.

In this study changes in expression of HM-type *N*-glycans were found which is in accordance with other studies (e.g., HM-type *N*-glycans increased in the progression from NE to CA) [13,16,30]. Surprisingly, when including the adenomatous areas, it appeared the HM-type *N*-glycans had the highest abundance in dysplastic areas; LGD and HGD areas, and were lower in CA. Moreover, by examining their distribution within the tissue sections, it could be shown that the progressive increase of HM was linked to the change from pre-invasive to invasive epithelial cells and not a result of the microenvironment surrounding the epithelial cells. Previous studies have shown increased levels of HM-type *N*-glycans in several diseases, including CRC and breast cancer [13,16,31] but it was never reported in precursor lesions. From the *N*-glycan biosynthesis point of view, increased HM suggests a precursor accumulation and/or an incomplete or limited *N*-glycan maturation, possibly due to shorter division/replication times (increased proliferation of the cells) [32]. As mentioned, Ki-67, a well-established immunohistochemical marker for cell proliferation, are highly expressed in cycling cells but strongly down-regulated in resting G0 cells [33]. The higher percentage of Ki-67 positive cells in the HGD areas was consistent with their increased proliferation, in comparison to the carcinomatous areas. This increase in proliferation is not correlated to an increase of cell density in HGD. Increased proliferation can be explained by the progressive enlargement of adenomas, a biological characteristic found in other pre-cancerous gastrointestinal lesions [26]. The relative decline in proliferative activity in the cancerous tissue, recalls the so-called “Gompertz effect” which embodies the fact that tumor cell growth rates decrease as a function of time [34,35]. This finding can potentially help to differentiate between pre-malignant and malignant areas.

Other changes in glycosylation were also observed, including a higher abundance of sialylated *N*-glycans during cancer progression. Especially the α2,3-linked sialic acids (CAm) *N*-glycans, which we were able to discern through the application of the linkage-specific sialic acid derivatization [36]. From our results, we found that the expression of CAm was higher in carcinoma but also in submucosal areas of the tissue. In a previous study of stage II CRC patient, we demonstrated that the cancer glycosylation signature, including the expression of CAm, spreads into the adjacent stroma at the tumor interface and that this interaction may play a role in patient survival [19]. This is consistent with other reports, in which increased levels of CAm *N*-glycans were associated with more aggressive forms of CRC in tissues and cell lines, and which has also been reported for other cancer [37,38,39,40,41]. Based on these literature reports, we can hypothesize that in our study the high CAm expression come from the cancer cells and not from other submucosal cells.

Regarding the α2,6-linked sialic acids (CD) *N*-glycans, a lower relative expression in CA areas was observed compared to the HGD regions. Increased expression of CD glycans have been reported in carcinomas of the colon, breast and cervix, choriocarcinomas, acute myeloid leukemia, and in some brain tumors [42,43,44,45,46,47,48,49]. Sata et al., detected α2,6-linked sialic acids with *Sambucus nigra I* lectin staining only in severe dysplasia and carcinoma of CRC tissues [50]. Dissimilarity in the results could be explained as later cancer stages and as well as different methods of analysis were used.

Our study revealed that the change of glycan antennarity through cancer progression is not as clear as expected. It has been widely shown in several cancer types that the increased expression of higher antennarity *N*-glycans (e.g., CA3, and CA4) is correlated with cell migration, tumor progression and invasion [51,52,53,54]. However, in our study we observed a lower expression in CA4 *N*-glycans in carcinoma compared with dysplastic region. This heterogeneity could be explained by two principal mechanisms underlying the tumor-associated alterations of carbohydrate structures, namely the so-called incomplete synthesis and neo-synthesis processes. The incomplete synthesis process is a consequence of the synthesis impairment of the complex-type *N*-glycans expressed in normal epithelial cells, which leads to the biosynthesis of incomplete structures. For instance via decreased expression or deletion of the enzymes establishing glycosidic linkages, the glycosyltransferase [55]. The incomplete synthesis process occurs more often in the early stage of cancer progression [56]. Conversely, neo-synthesis, commonly observed in more advanced stages of cancer, refers to a direct association between glycan alterations and the genetic mechanism for malignant transformation of cells, as seen in transcriptional induction of *MGAT5* (GlcNAc transferase-V responsible for the synthesis of triantennary glycan) by v-*src*, H-*ras*, and v-*fps* genes [57]. Based on these two principals (neo-synthesis and incomplete synthesis), our observation could be correlated to the normal synthesis of the CA3 *N*-glycans in the epithelium and dysregulated expression of CA3 due to deregulation of the responsible glycosyltransferase in cancer.

We investigated whether there are glycosylation markers for CRC with and without LNM as the presence of LNM influences the treatment options and consequently the patient’s prognosis, morbidity, and mortality. However, as this is still a rare occurrence in patient with early stage CRC, only few cases could be used for the analysis. Furthermore, at the time of the analysis there was only 2–3 years follow-up of the patient available, so not enough data. We still observed that one fully sialylated triantennary complex-type *N*-glycan exhibited slightly higher expression in the LNM+ group. This finding is in line with literature as this *N*-glycan contains sialylation and has high antennarity which are both features associated with advanced cancer as discussed above. As the number of samples is low, analysis of a larger cohort is needed to verify if this finding could be used as a marker for LNM staging in CRC.

## 5. Conclusions

Our analysis provides new insights in the *N*-glycosylation signatures featuring each step of the progression from adenoma to pT1 CRC. Still little is known in literature regarding this early cancer and our study shows an increase in HM-type *N*-glycans which is linked to an increased proliferation of the cells. While this increase from normal epithelium to cancer is already known from literature, we demonstrated that the highest increase was shown in the dysplastic regions of the pre-malignant lesion. This finding could help pathological evaluation of cancer stages by distinguishing pre-malignant from malignant lesion. Other findings include changes in the cancer invasive front which match our previous observation in pT2 CRC.

## Figures and Tables

**Table 1:** Summary of the analyzed cohort.

Patient	21
Gender	
Male (%)	17 (81)
Female (%)	3 (14)
Unknown (%)	1 (5)
Median age (min–max)	66 (57–81)
Topography	
Rectum	4
Rectosigmoid	2
Sigmoid	13
Descending	1
Transverse	1
Morphology	
Sessile	11
Pedunculated	1
Na	9
Differentiation	
Well /medium	9
Medium	2
Na	10
Lymph-node metastasis	
LNM0	4
LNM+	5
Na	12
Diameter (avg cm (min–max))	1.68 (0.6–2.4)
Depth (avg mm (min–max))	4.18 (2–9)
p53	
Mutant	4
Unknown	17
Lymphovascular invasion	
No	4
Yes	6
Na	11
Number of tissues with	
Adjacent Normal colon epithelium	20
Low-grade dysplasia	16
High-grade dysplasia	7
Carcinoma	10

## Data Availability

Data are contained within the article.

## Supplementary Materials

The following are available online at https://www.mdpi.com/article/10.3390/cancers14061552/s1, Figure S1: Annotated H&E stained CRC tissues; Figure S2: Relative changes of glycans and derived traits in early CRC development; Figure S3: Ki-67 staining in different morphological stages of CRC progression; Figure S4: Glycomic changes between early stages CRC morphologies; Figure S5: Glycan change between lymph node metastasis (LNM) status; Table S1: Derived trait calculation and explanation; Table S2: Feature selection by Kruskal-Wallis test (KW); Table S3: Overview of the 58 *N*-glycans detected by MALDI-MSI; File S1: Histological annotation of the cohort.
