# Supplementary material for: High-Mannose N-Glycans as Malignant Progression Markers in Early-Stage Colorectal Cancer
Source: Cancers (Basel). 2022 Mar 18;14(6):1552. doi: 10.3390/cancers14061552 (PMC8945895; doi:10.3390/cancers14061552)
Supplement: Supplementary file 1 [file cancers-14-01552-s001.zip › supplementary figures.pdf]

**Supplementary information to**

# High-Mannose *N*-Glycans as Malignant Progression Markers in Early-Stage Colorectal Cancer

**Fanny Boyaval [1,2], Hans Dalebout [2], René Van Zeijl [2], Wenjun Wang [2], Arantza Fariña-Sarasqueta [3], Guinevere S. M. Lageveen-Kammeijer [2], Jurjen J. Boonstra [4], Liam A. McDonnell [5], Manfred Wuhrer [2], Hans Morreau [1,*] and Bram Heijs [2,*]**

1 Department of Pathology, Leiden University Medical Center, Albinusdreef, 2333 ZA Leiden,
The Netherlands; f.f.m.boyaval@lumc.nl

2 Center for Proteomics & Metabolomics, Leiden University Medical Center, Albinusdreef, 2333 ZA Leiden, The Netherlands; h.dalebout@lumc.nl (H.D.); r.j.m.van_zeijl@lumc.nl (R.V.Z.); w.wang@lumc.nl (W.W.); g.s.m.kammeijer@lumc.nl (G.S.M.L.-K.); m.wuhrer@lumc.nl (M.W.)

3 Department of Pathology, Cancer Center Amsterdam, Amsterdam University Medical Centers, University of Amsterdam, Meibergdreef 9, 1105 AZ Amsterdam, The Netherlands; a.farina@amsterdamumc.nl

4 Department of Gastroenterology and Hepatology, Leiden University Medical Centre, 2300 RC Leiden,
The Netherlands; j.j.boonstra@lumc.nl

5 Fondazione Pisana per la Scienza ONLUS, Via Ferruccio Giovannini, 56017 San Giuliano Terme, Italy; l.a.mcdonnell@outlook.com

* Correspondence: j.morreau@lumc.nl (H.M.); b.p.a.m.heijs@lumc.nl (B.H.)

## Supplementary Figure S1:

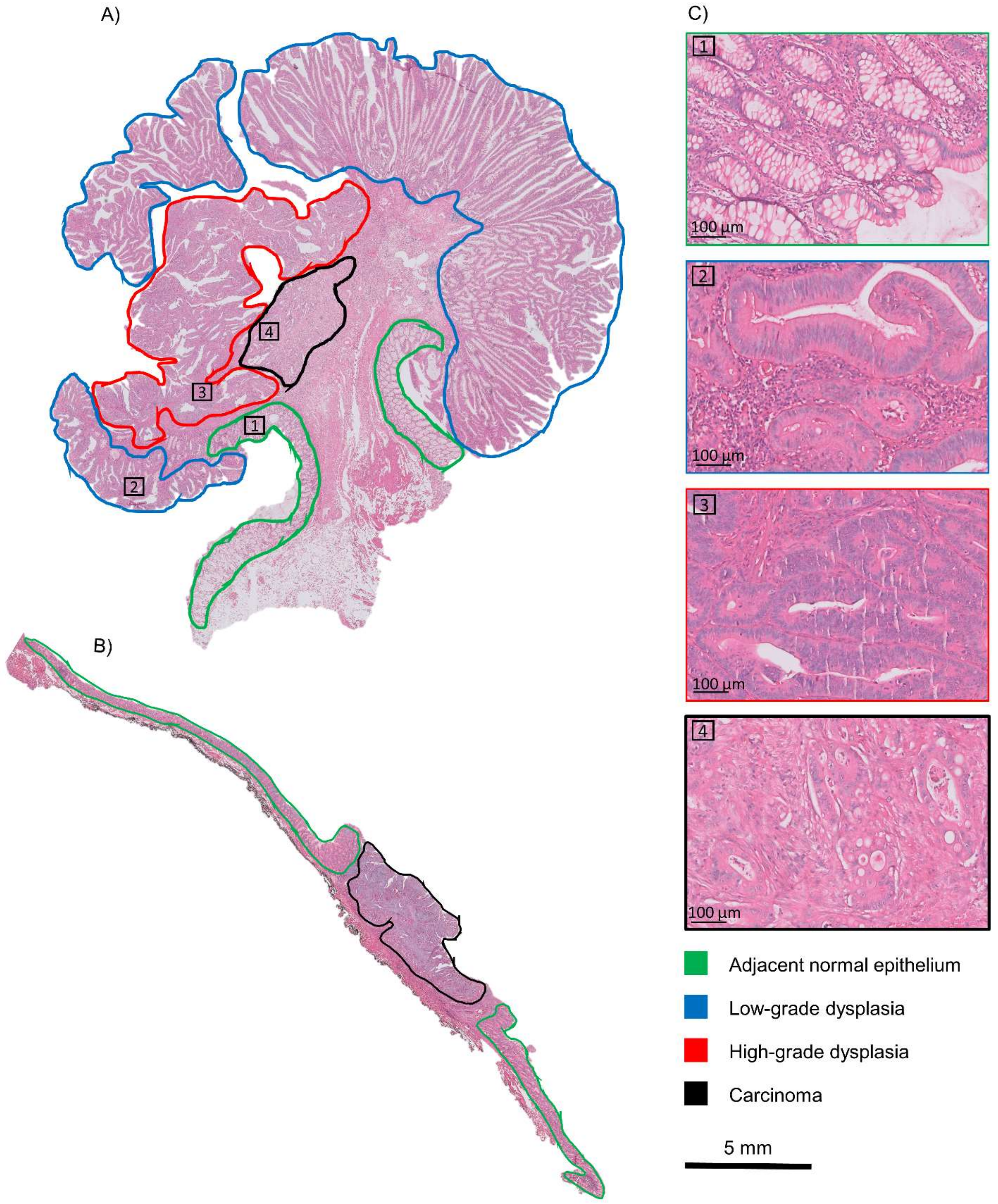


**Figure S1. Annotated H&E stained CRC tissues. (A)** & **(B)** Examples of post-MSI H&E stained human pT1 CRC tissues with annotations of morphological regions of interest. **(C)** Zoomed images (20x magnification) of different morphological areas annotated in the tissue (A).

**Supplementary Figure S2:**

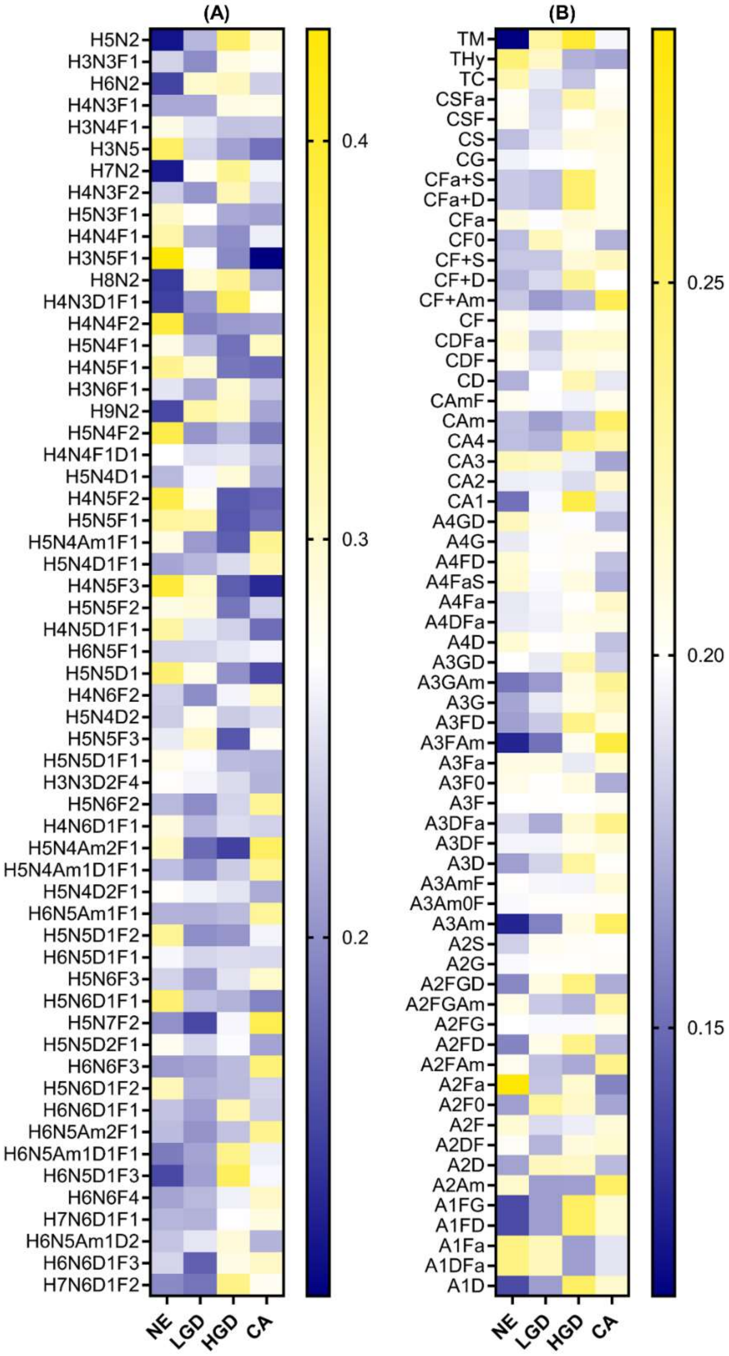


**Figure S2. Relative changes of glycans and derived traits in early CRC development.** Heatmap of the relative abundances of **(A)** individual *N*-glycan and **(B)** derived traits. The relative intensities have been averaged per morphology and scaled by the sum of each row for better visualization. NE = adjacent normal epithelium, LGD = low-grade dysplasia, HGD = high-grade dysplasia, CA = carcinoma. H: hexose, N: *N*-acetyl hexosamine, Am: α2,3-linked *N*-acetyl neuraminic acid, D: α2,6-linked *N*-acetyl neuraminic acid F: fucose.

**Supplementary Figure S3:**

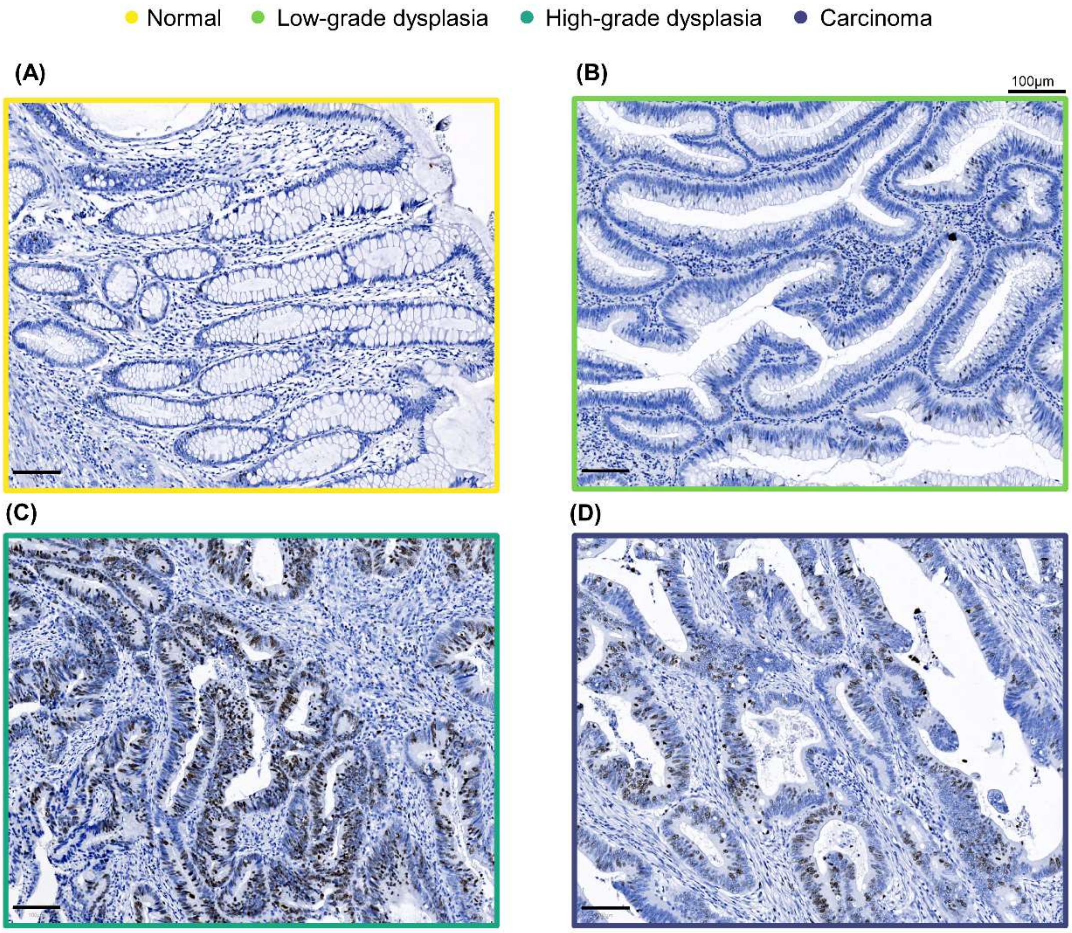


**Figure S3. Ki-67 staining in different morphological stages of CRC progression.** Immunohistochemistry of Ki-67 in selected regions of **(A)** normal adjacent epithelium (NE), **(B)** low-grade dysplasia (LGD), **(C)** high-grade dysplasia (HGD) and **(D)** carcinoma (CA).

**Supplementary Figure S4:**

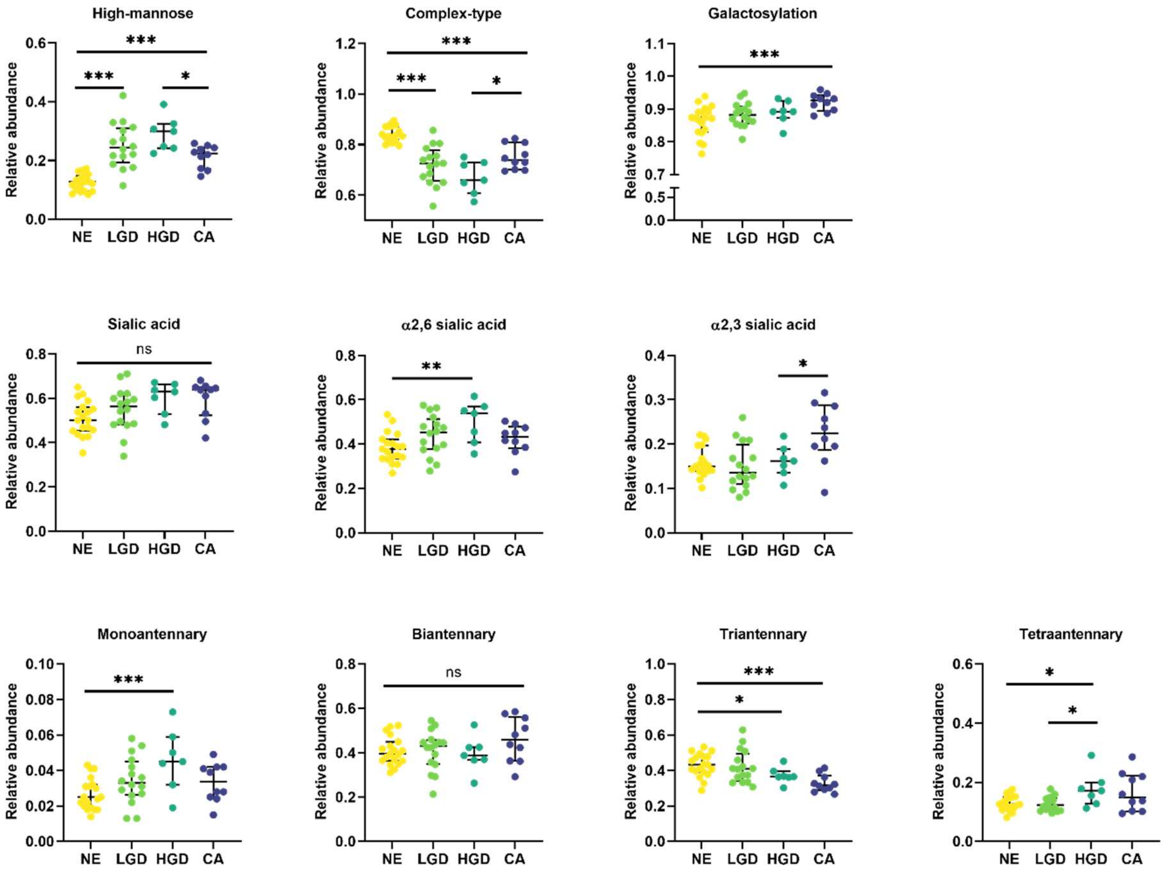


**Figure S4. Glycomic changes between early stages CRC morphologies.** Scatter plots of selected derived traits representing the median with interquartile range of the different morphologies per patient. NE = adjacent normal epithelium, LGD = low-grade dysplasia, HGD = high-grade dysplasia, CA = carcinoma. * = p-value ≤ 0.05, ** = p-value ≤ 0.01, *** = p-value ≤ 0.001, ns = not significant.

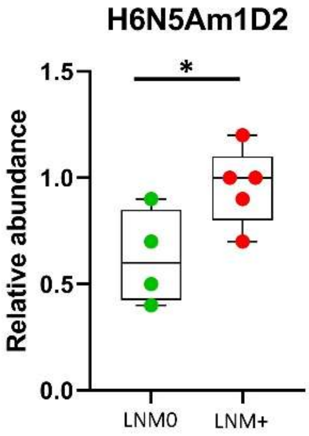


**Figure S5. Glycan change between lymph node metastasis (LNM) status.** Scatter plots of the glycan H6N5Am1D2 representing the median with interquartile range of LNM negative (LNM0) and positive (LNM+) patients. * = p-value $\leq 0.05$
