# Supplementary material for: High-Mannose N-Glycans as Malignant Progression Markers in Early-Stage Colorectal Cancer
Source: Cancers (Basel). 2022 Mar 18;14(6):1552. doi: 10.3390/cancers14061552 (PMC8945895; doi:10.3390/cancers14061552)
Supplement: Supplementary file 1 [file cancers-14-01552-s001.zip › Supplementary-Document-D1.pdf]

Legend:

- Normal epithelium
- Low-grade dysplasia
- High-grade dysplasia
- Cancer
- Pseudoinvasion
- High-grade dysplasia or cancer

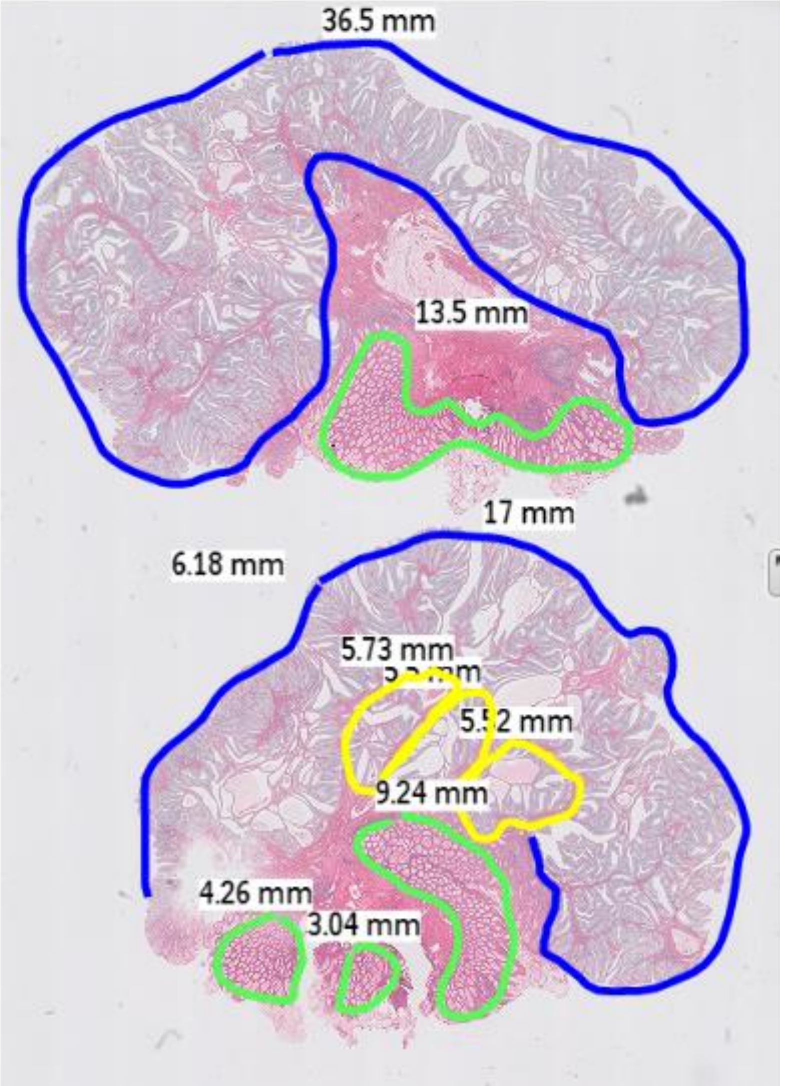
36.5 mm
13.5 mm
17 mm
6.18 mm
5.73 mm
5.52 mm
9.24 mm
4.26 mm
3.04 mm

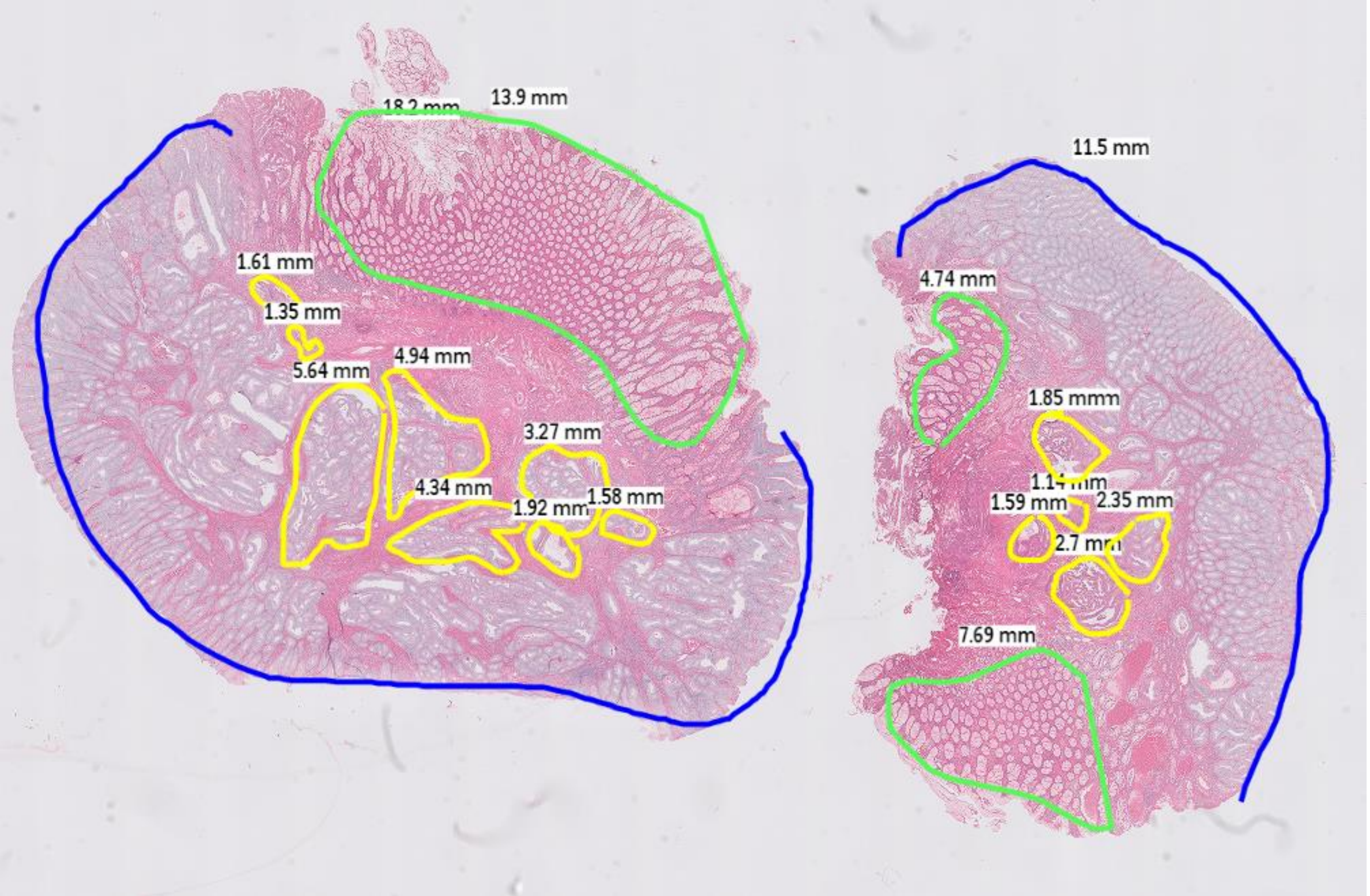
18.2 mm
13.9 mm
11.5 mm
1.61 mm
1.35 mm
4.74 mm
4.94 mm
5.64 mm
1.85 mmm
3.27 mm
1.14 mm
4.34 mm
1.58 mm
1.92 mm
1.59 mm
2.35 mm
2.7 mm
7.69 mm

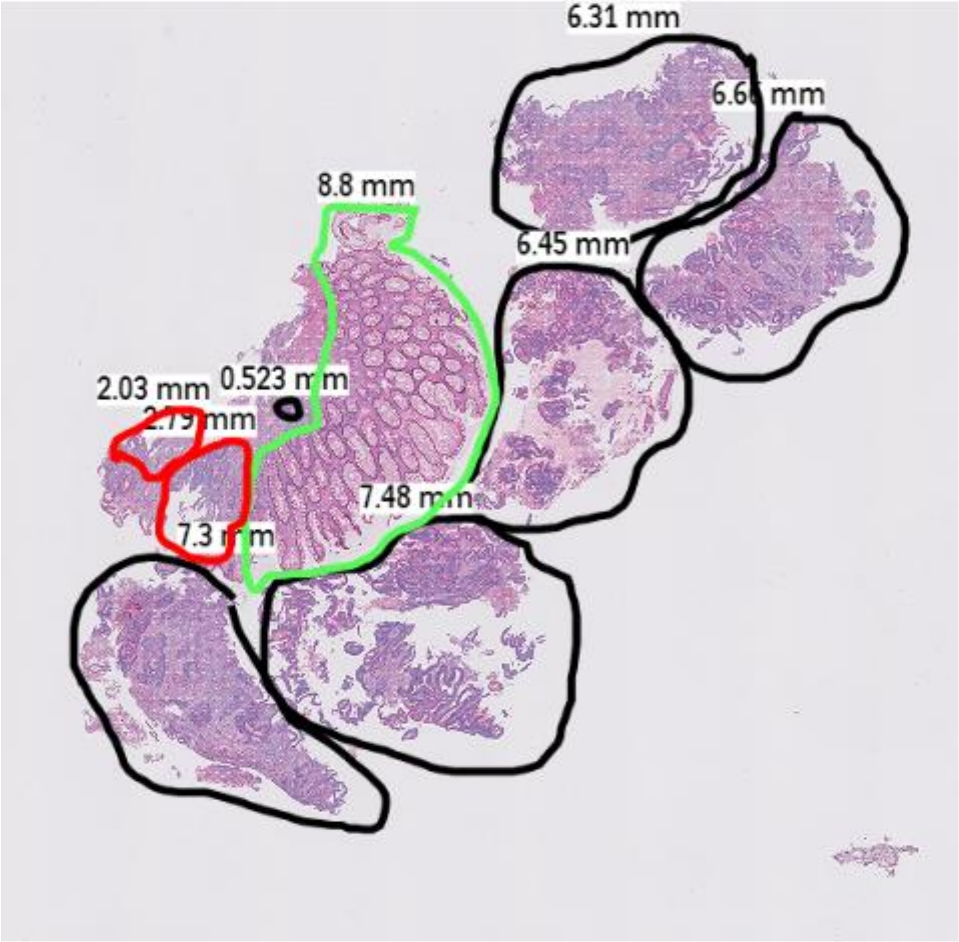
6.31 mm
8.8 mm
6.45 mm
2.03 mm
0.523 mm
2.79 mm
7.48 mm
7.3 mm

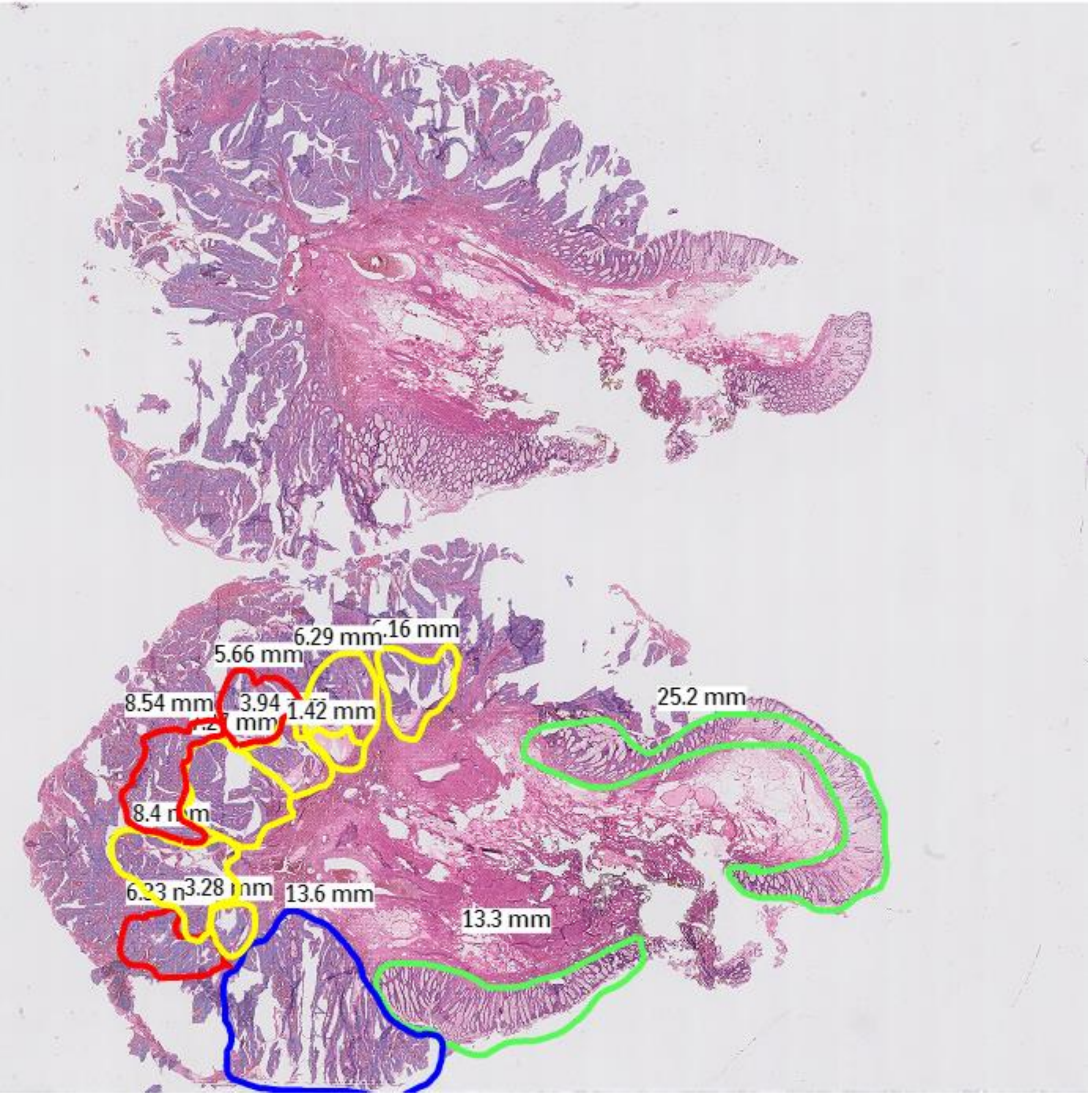
6.29 mm
5.66 mm
8.54 mm
1.42 mm
25.2 mm
3.28 mm
13.6 mm
13.3 mm

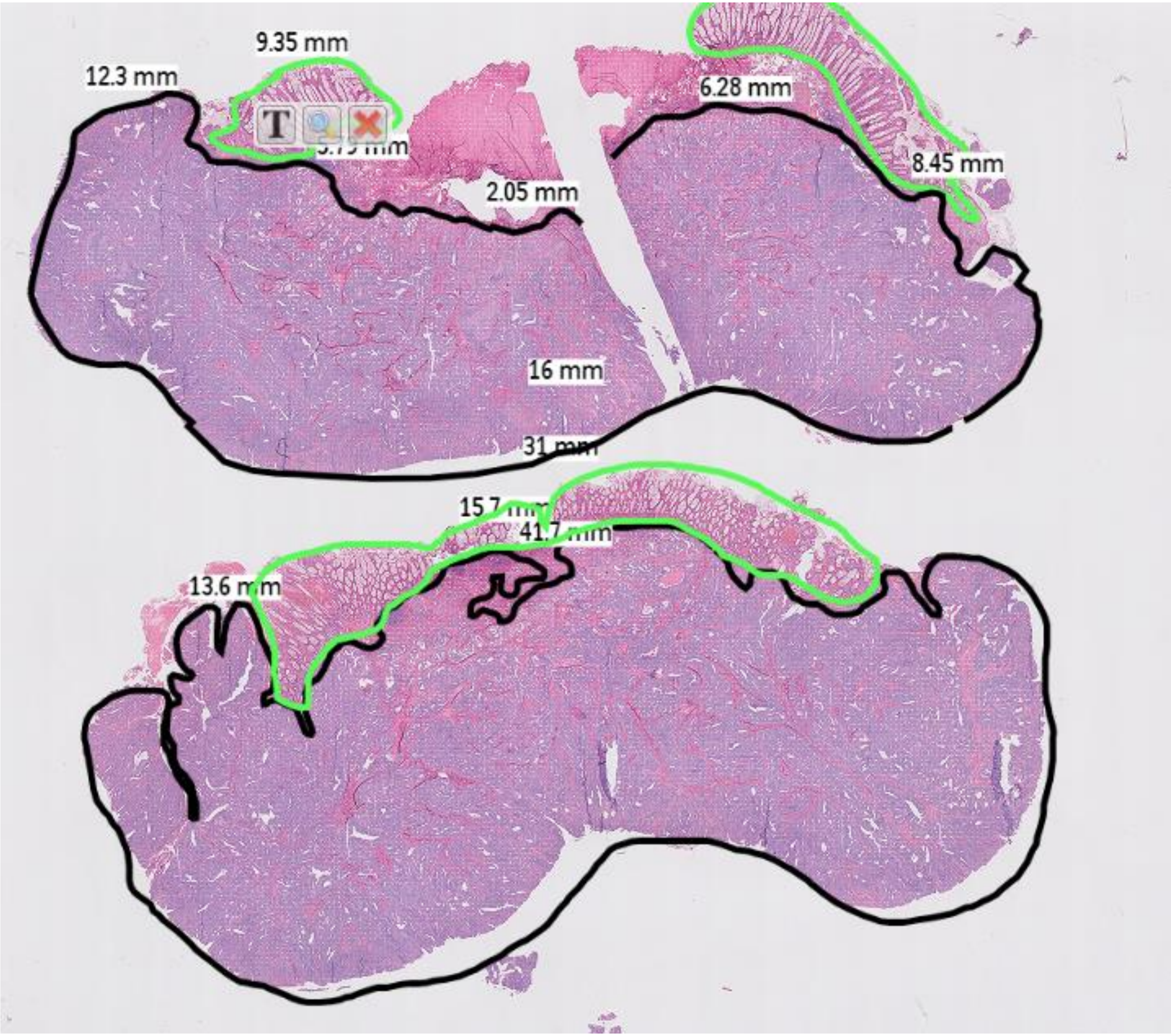

9.35 mm
12.3 mm
6.28 mm
2.05 mm
8.45 mm
16 mm
31 mm
15.7 mm
41.7 mm
13.6 mm

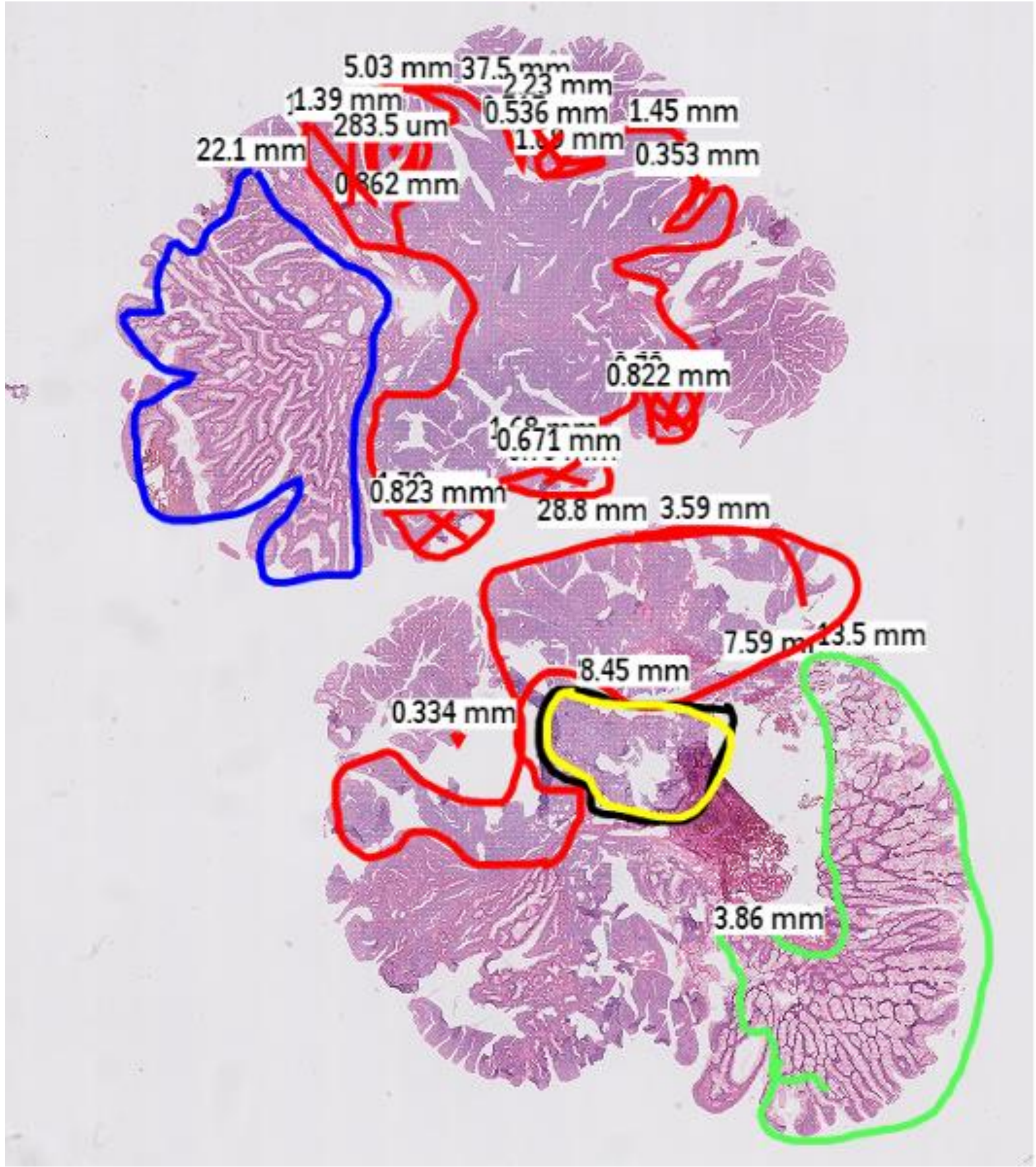
5.03 mm
37.5 mm
2.23 mm
1.39 mm
0.536 mm
1.45 mm
283.5 um
22.1 mm
0.353 mm
0.862 mm
0.822 mm
0.671 mm
0.823 mm
28.8 mm
3.59 mm
13.5 mm
8.45 mm
0.334 mm
3.86 mm

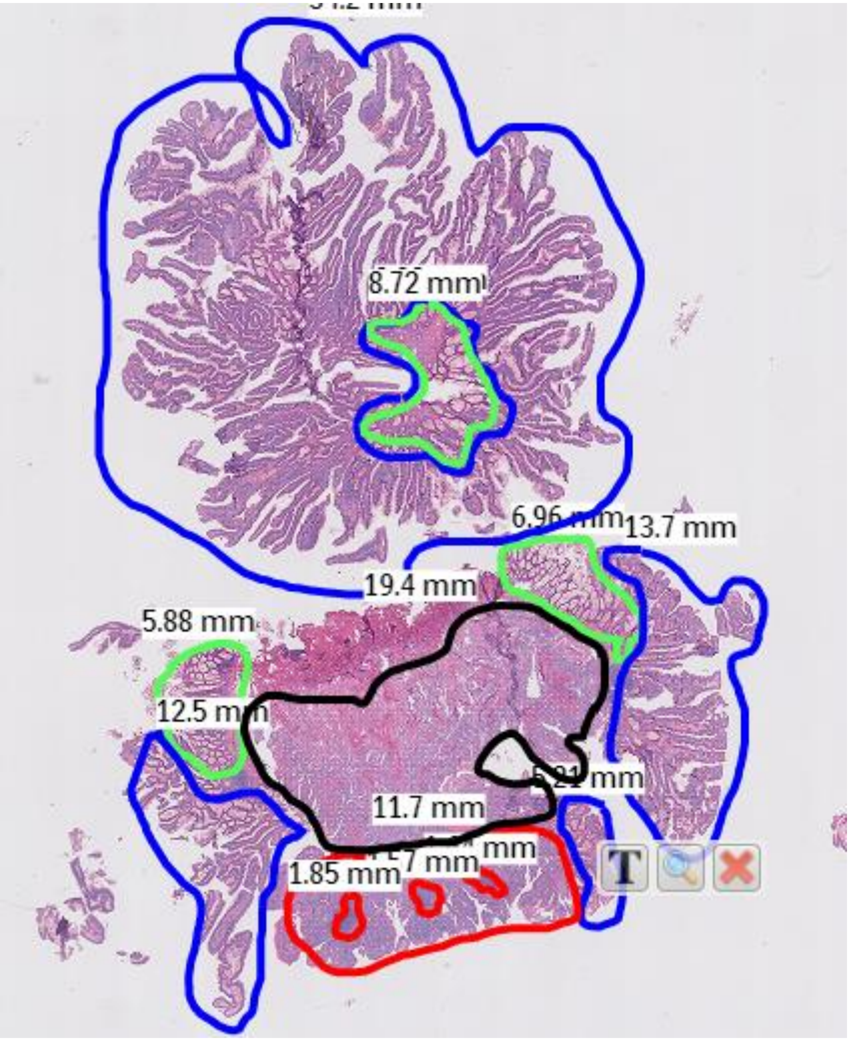
8.72 mm
6.96 mm
13.7 mm
19.4 mm
5.88 mm
12.5 mm
11.7 mm
1.85 mm

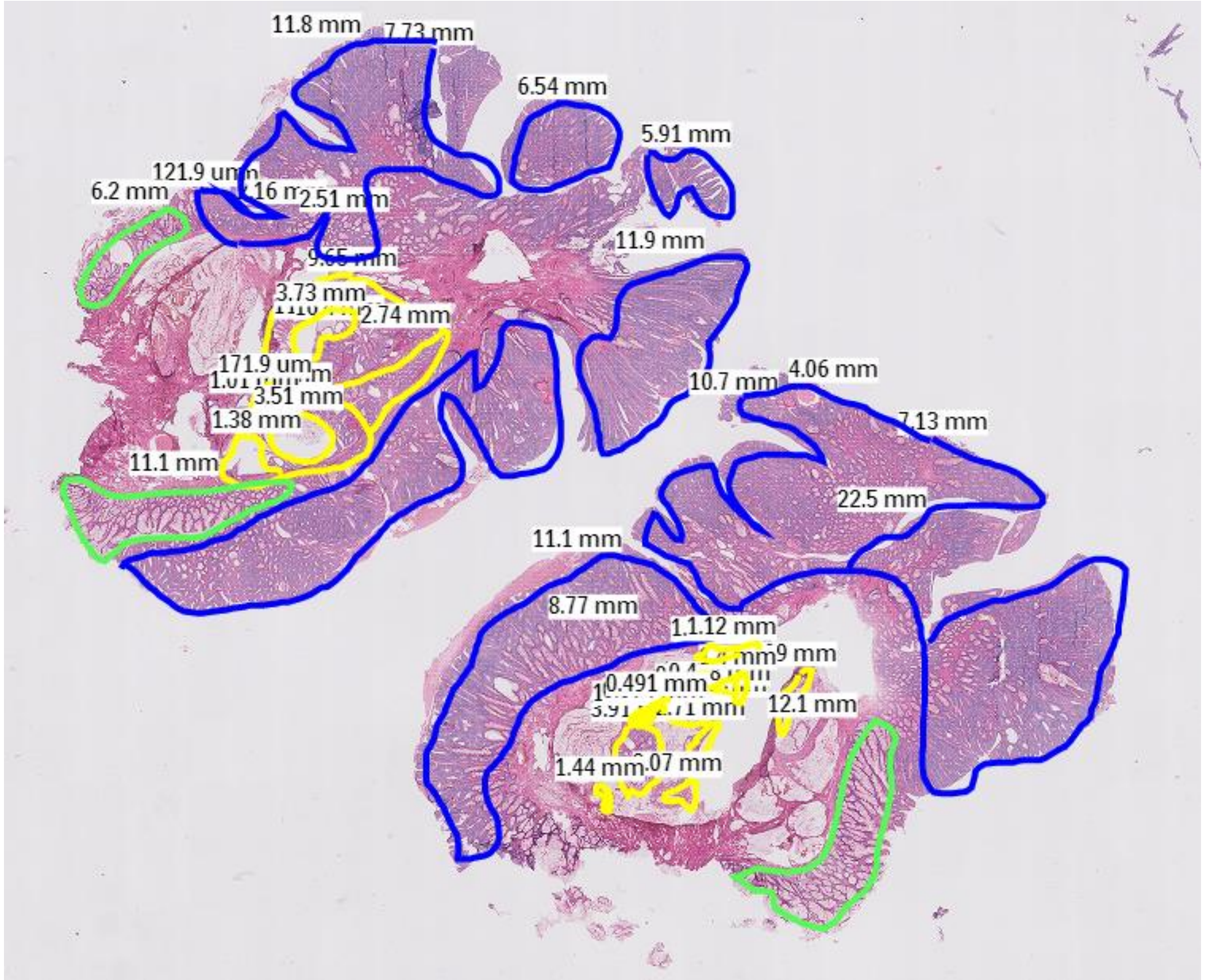
11.8 mm
7.73 mm
6.54 mm
5.91 mm
121.9 um
6.2 mm
2.51 mm
11.9 mm
3.73 mm
2.74 mm
171.9 um
3.51 mm
1.38 mm
11.1 mm
10.7 mm
4.06 mm
7.13 mm
22.5 mm
11.1 mm
8.77 mm
0.491 mm
12.1 mm
1.44 mm

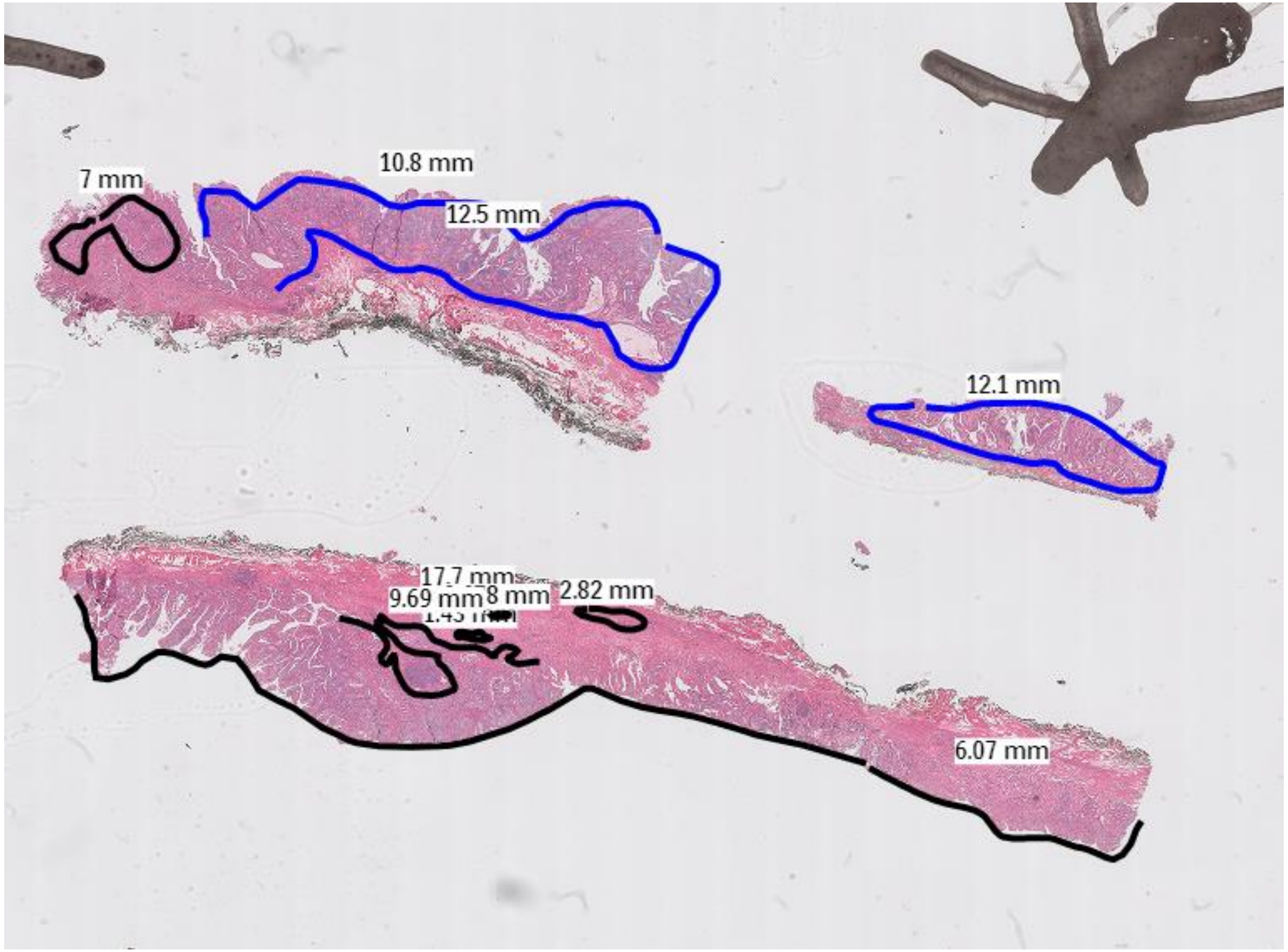

7 mm
10.8 mm
12.5 mm
12.1 mm
17.7 mm
9.69 mm
8 mm
2.82 mm
6.07 mm

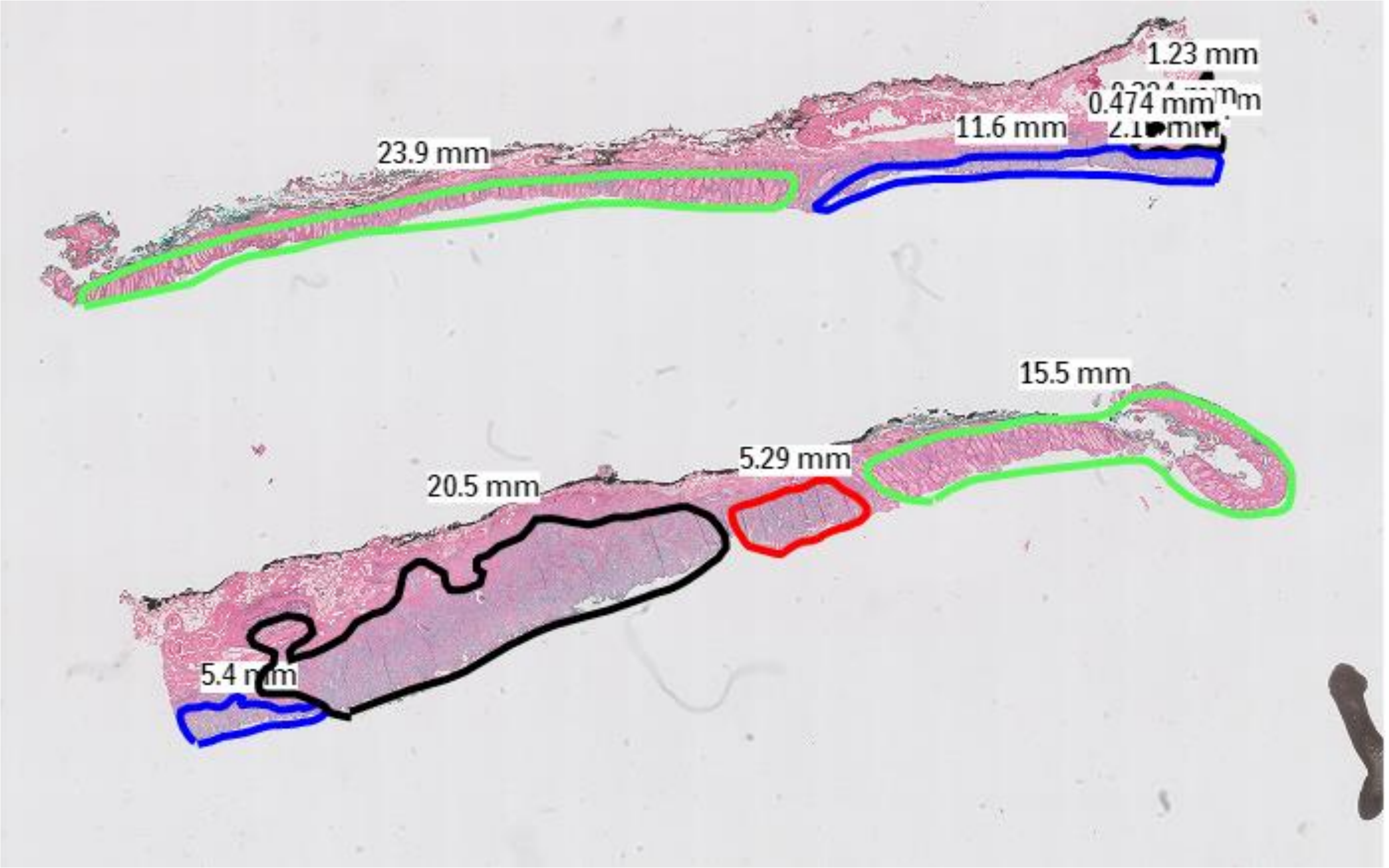
1.23 mm
0.474 mm
11.6 mm
23.9 mm
15.5 mm
5.29 mm
20.5 mm
5.4 mm

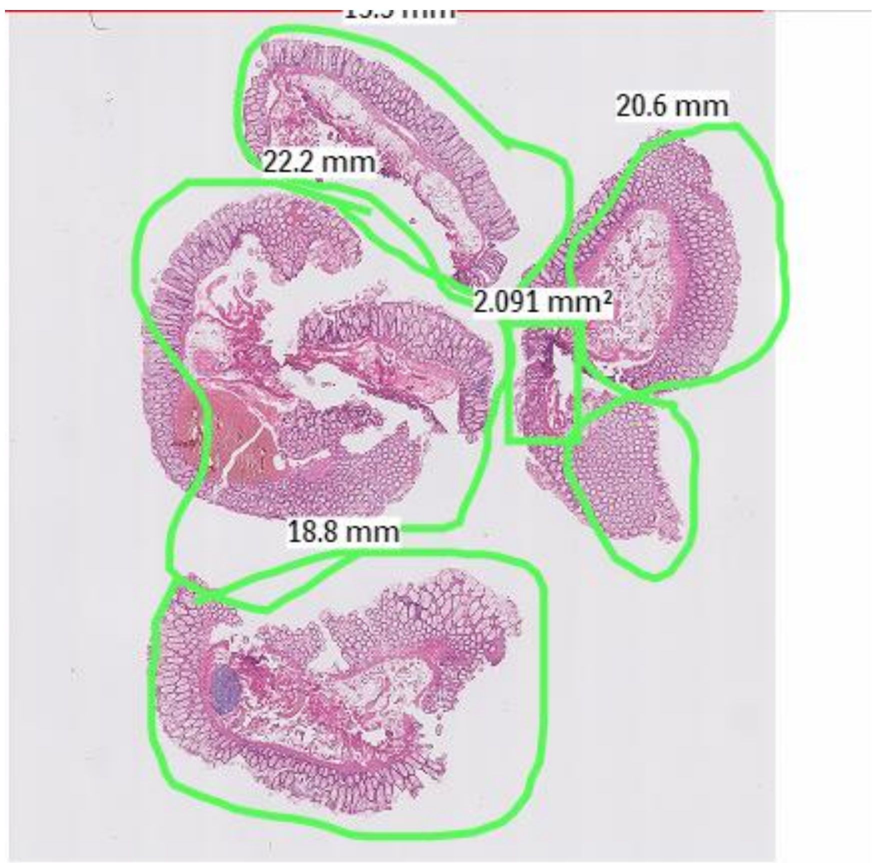
22.2 mm
20.6 mm
2.091 mm²
18.8 mm

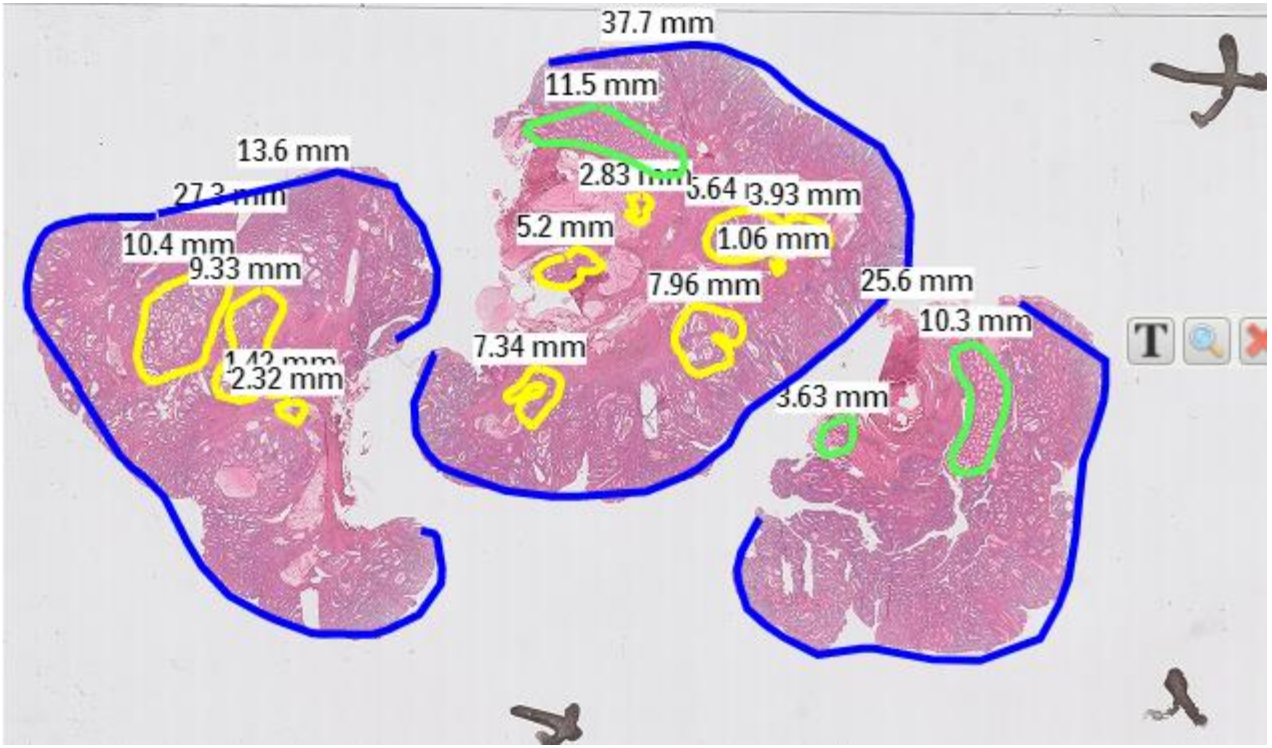
37.7 mm
11.5 mm
13.6 mm
27.3 mm
3.93 mm
5.2 mm
1.06 mm
10.4 mm
9.33 mm
7.96 mm
25.6 mm
10.3 mm
2.32 mm
7.34 mm
3.63 mm

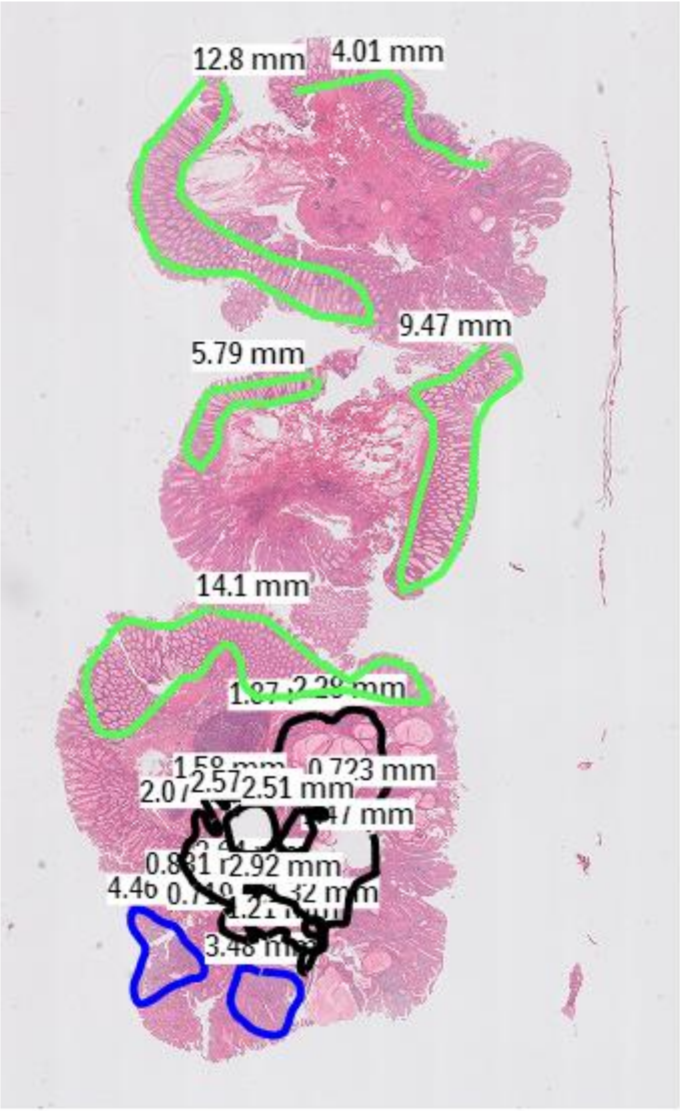
12.8 mm
4.01 mm
5.79 mm
9.47 mm
14.1 mm
0.723 mm
2.51 mm
2.92 mm

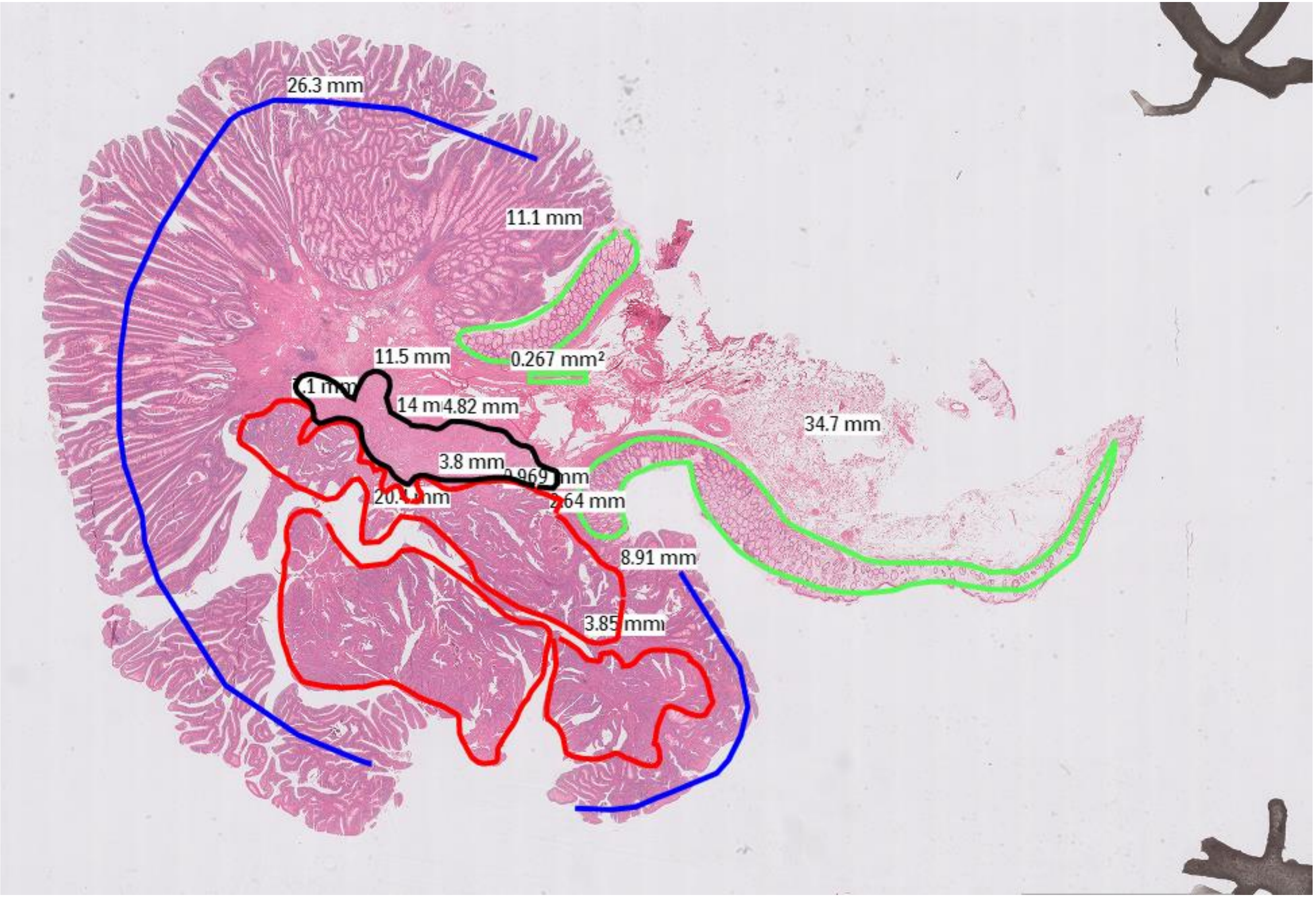
26.3 mm
11.1 mm
11.5 mm
0.267 mm²
4.82 mm
34.7 mm
3.8 mm
0.969 mm
2.64 mm
8.91 mm
3.85 mm

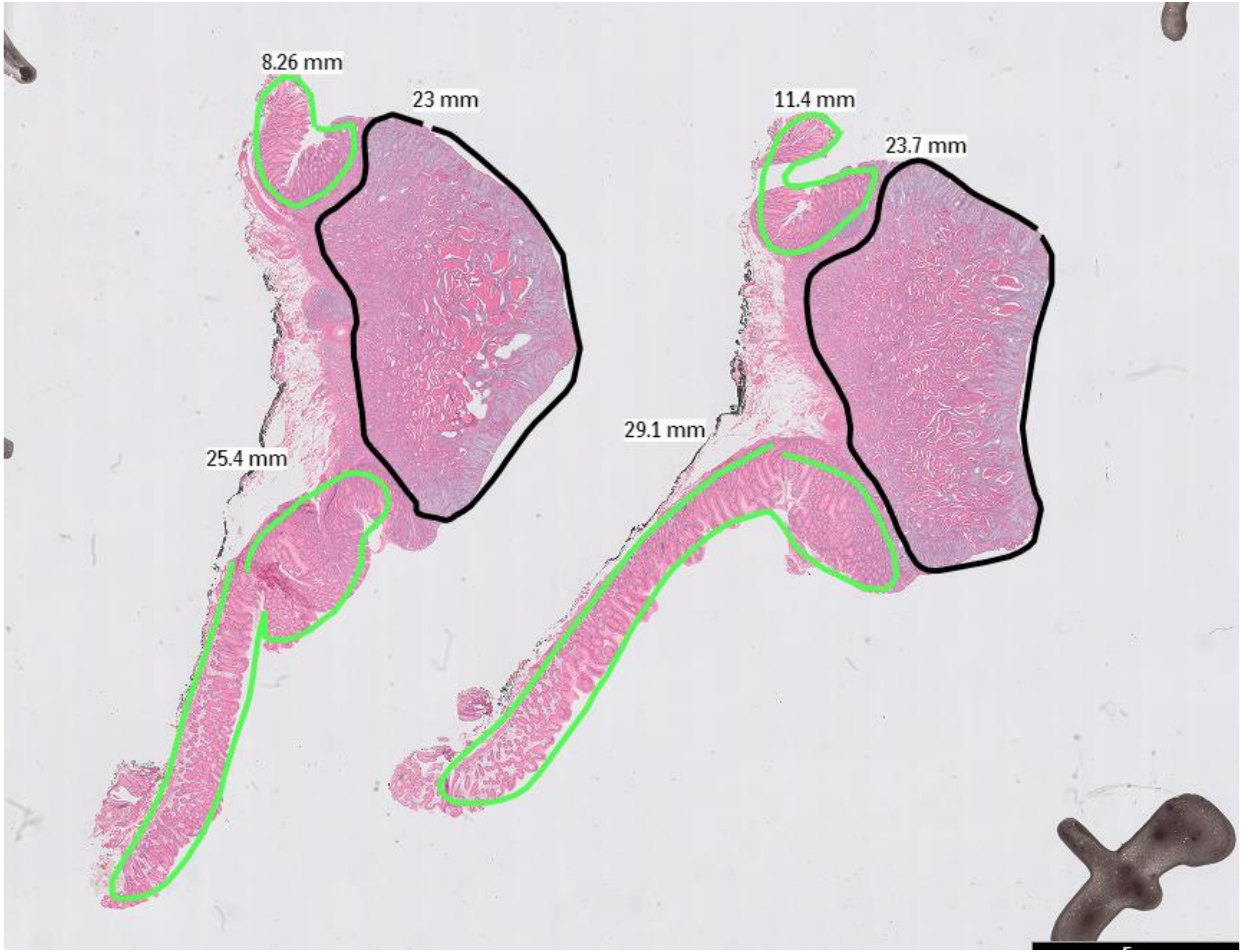
8.26 mm
23 mm
11.4 mm
23.7 mm
25.4 mm
29.1 mm

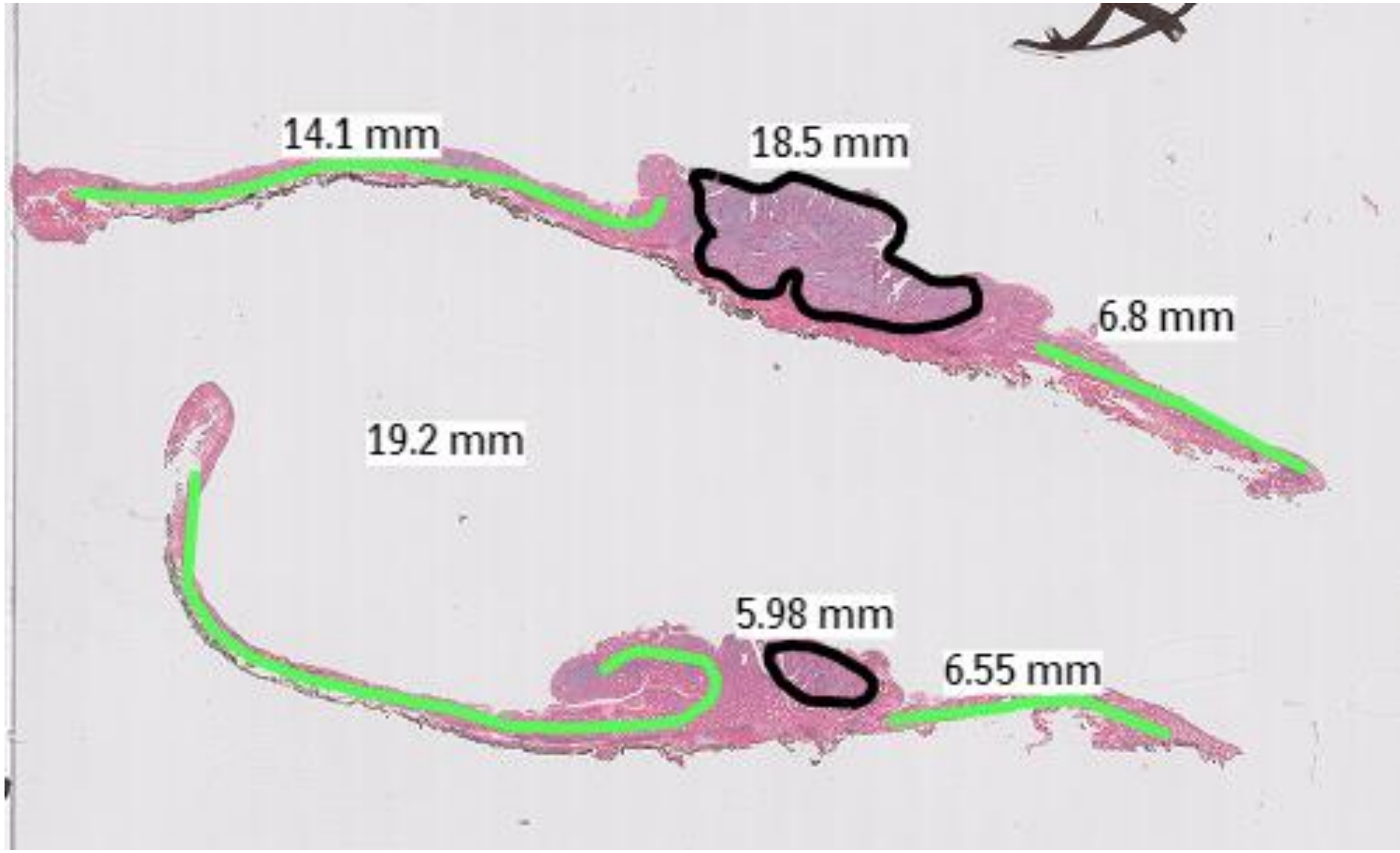
14.1 mm
18.5 mm
6.8 mm
19.2 mm
5.98 mm
6.55 mm

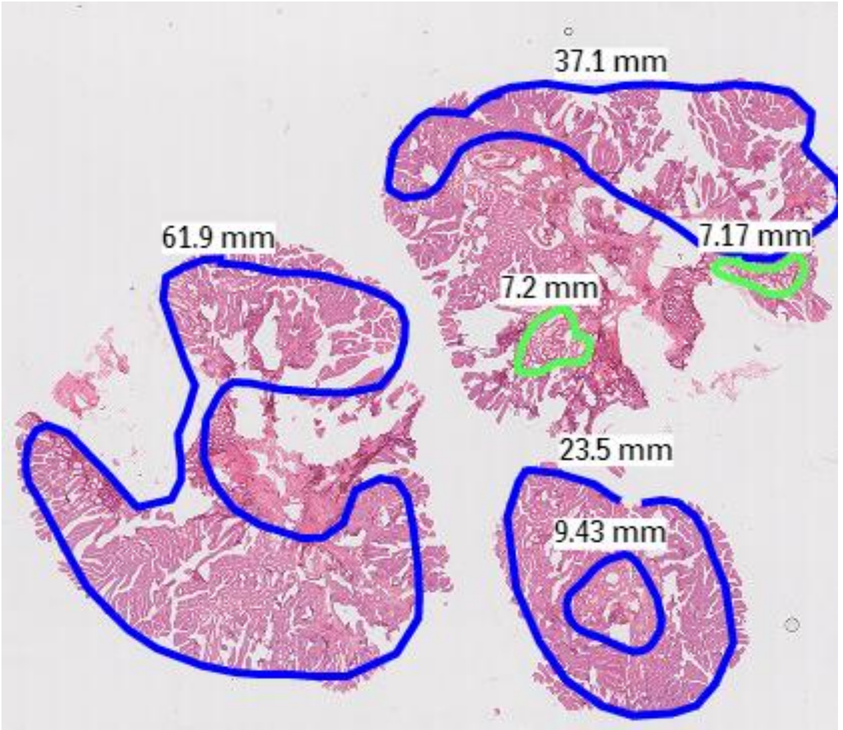
37.1 mm
61.9 mm
7.17 mm
7.2 mm
23.5 mm
9.43 mm

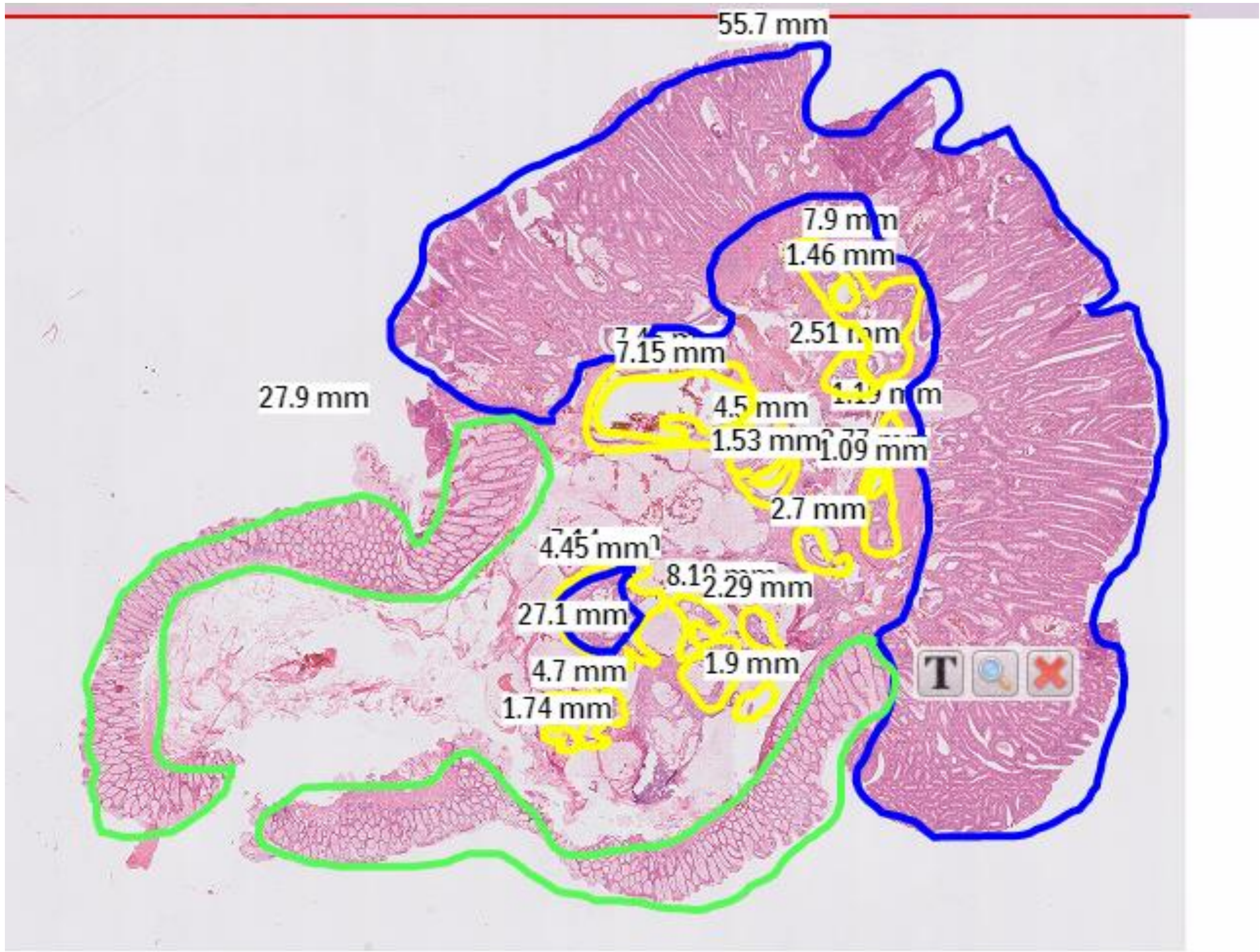
55.7 mm
7.9 mm
1.46 mm
2.51 mm
7.15 mm
27.9 mm
4.5 mm
1.53 mm
1.09 mm
2.7 mm
4.45 mm
2.29 mm
27.1 mm
4.7 mm
1.9 mm
1.74 mm

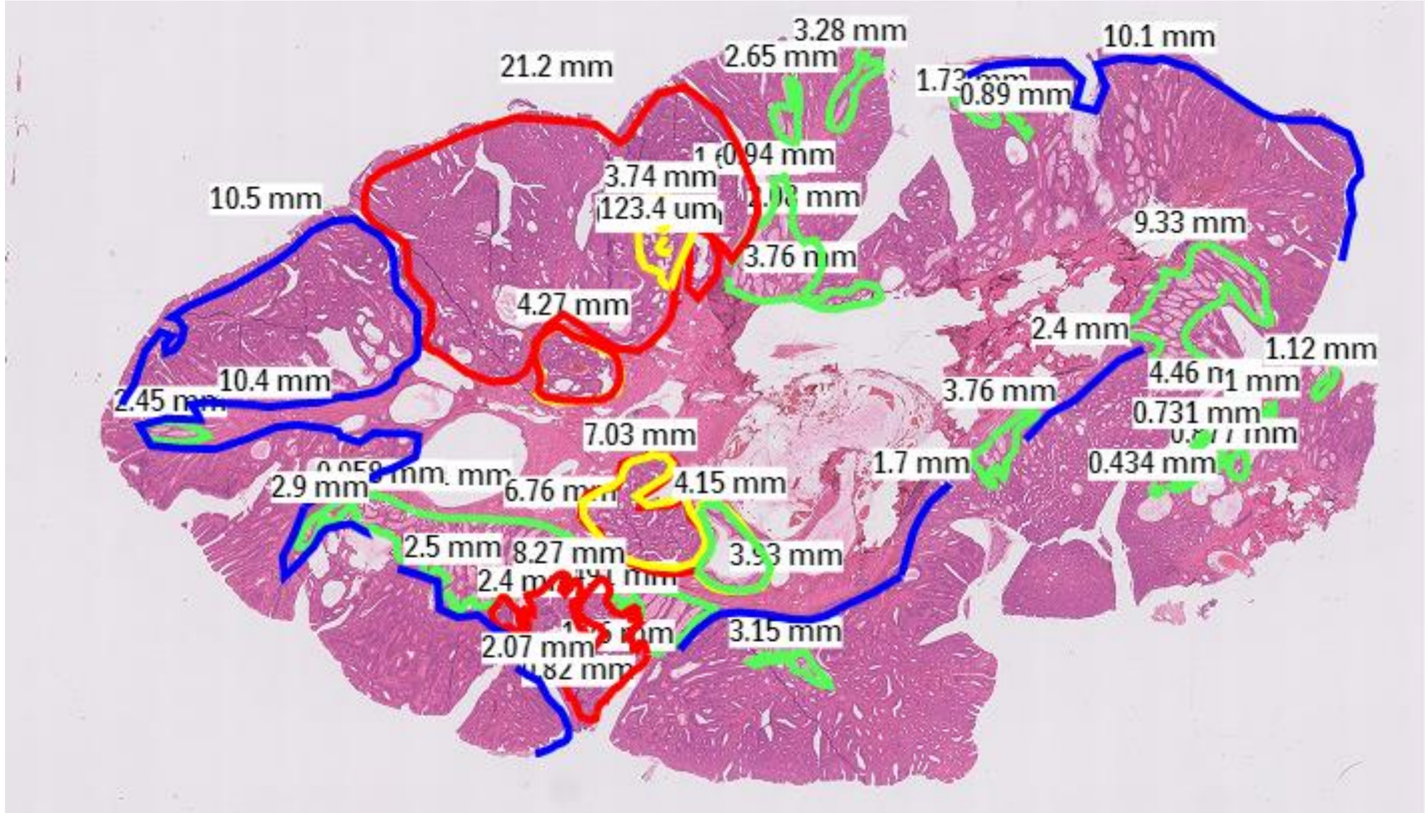

21.2 mm
3.28 mm
2.65 mm
10.1 mm
0.89 mm
10.5 mm
3.74 mm
123.4 um
0.94 mm
3.76 mm
9.33 mm
4.27 mm
2.4 mm
1.12 mm
10.4 mm
3.76 mm
0.731 mm
7.03 mm
1.7 mm
0.434 mm
2.9 mm
4.15 mm
2.5 mm
8.27 mm
3.15 mm
2.07 mm

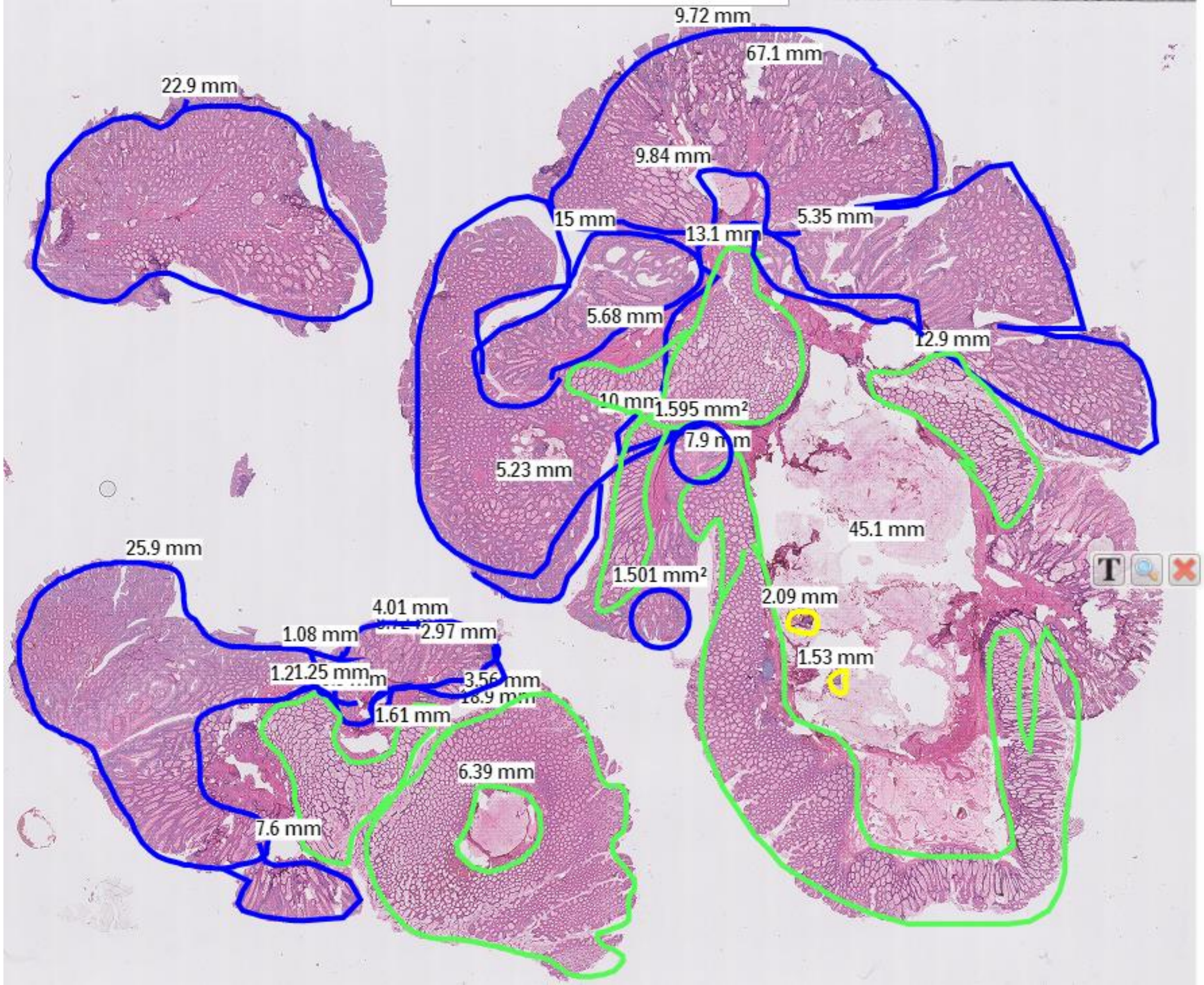
9.72 mm
67.1 mm
22.9 mm
9.84 mm
15 mm
13.1 mm
5.35 mm
5.68 mm
12.9 mm
1.595 mm^2
5.23 mm
45.1 mm
25.9 mm
1.501 mm^2
2.09 mm
4.01 mm
1.08 mm
2.97 mm
1.53 mm
3.56 mm
1.61 mm
6.39 mm
7.6 mm

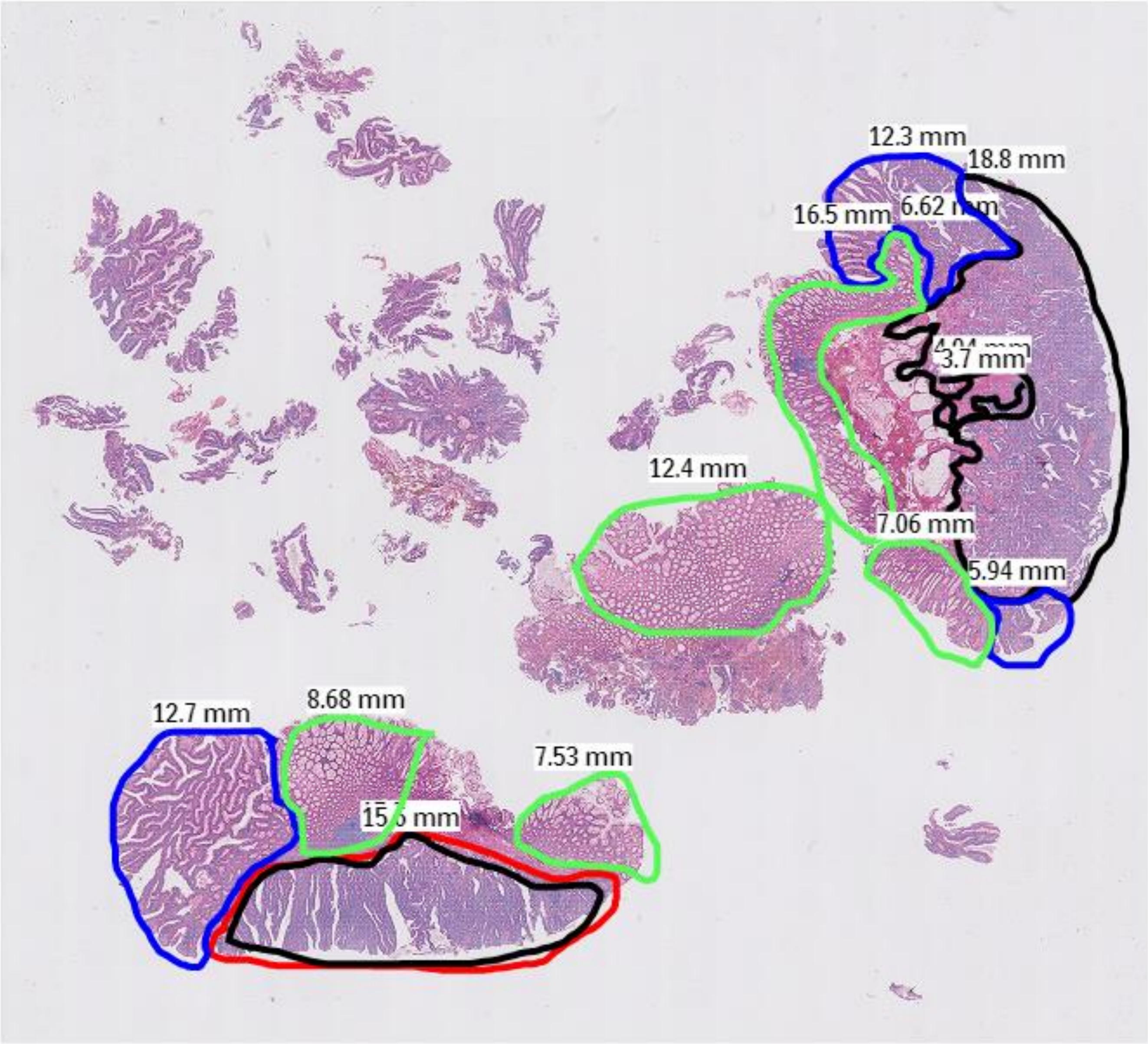
12.3 mm
18.8 mm
16.5 mm
3.7 mm
12.4 mm
7.06 mm
5.94 mm
12.7 mm
8.68 mm
7.53 mm
